# 2D Carbon Phosphide for Trapping Sulfur in Rechargeable Li–S Batteries: Structure Design and Interfacial Chemistry

**DOI:** 10.1021/acsami.4c15372

**Published:** 2024-12-16

**Authors:** Nabil Khossossi, Mohammed Lemaalem, Talha Zafer, Abdelfattah Mahmoud, Poulumi Dey

**Affiliations:** † Department of Materials Science and Engineering, Faculty of Mechanical Engineering, Delft University of Technology, Mekelweg 2, 2628 CD Delft, The Netherlands; ‡ Department of Chemical Engineering, University of Illinois Chicago, Chicago, Illinois 60608, United States; ¶ Materials Science Division, Argonne National Laboratory, Lemont, Illinois 60439, United States; § Vocational School of Health Services, Sakarya University, 54050 Sakarya, Turkey; ∥ GREENMAT, CESAM, Institute of Chemistry B6, University of Liège, 4000 Liège, Belgium

**Keywords:** 2D CP_3_, Electrochemical properties, Shuttle effect, Lithium polysulfides, Organic electrolyte, DFT, MD

## Abstract

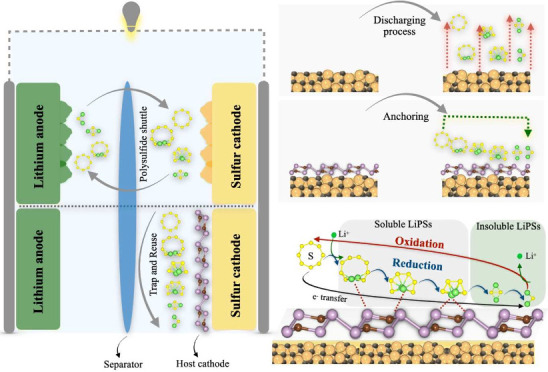

Rechargeable lithium–sulfur batteries (LiSBs) assembled with earth-abundant and safe Li anodes are less prone to form dendrites on the surface, and sulfur-containing cathodes offer considerable potential for achieving high energy densities. Nevertheless, suitable sulfur host materials and their interaction with electrolytes are at present key factors that retard the commercial introduction of these batteries. Here we propose a two-dimensional metallic carbon phosphorus framework, namely, 2D CP_3_, as a promising sulfur host material for inhibiting the shuttle effect and improving electronic conductivity in high-performance Li–S batteries. The good electrical conductivity of CP_3_ eliminates the insulating nature of most sulfur-based electrodes. The dissolution of lithium polysulfides (LiPSs) into the electrolyte is largely prevented by the strong interaction between CP_3_ and LiPSs. In addition, the deposition of Li_2_S on CP_3_ facilitates the kinetics of the LiPS redox reaction. Therefore, the use of CP_3_ for Li–S battery cathodes is expected to suppress the LiPS shuttle effect and to improve the overall performance, which is ideal for the practical application of Li–S batteries.

## 1 Introduction

In the evolving landscape of electric vehicle technology, substantial research has been directed toward enhancing the performance of rechargeable batteries, with a particular focus on augmenting energy density, extending cycle life, and maintaining operational efficiency at elevated temperatures.^1,2^ Lithium-ion batteries, which rely on the Li-intercalation mechanism, are among the most prevalent in the current market. Despite their widespread use, challenges such as safety and efficient operation, particularly related to capacity fading due to various degradation mechanisms like the formation and breakdown of Solid-Electrolyte-Interphase (SEI), remain significant.^3−6^ Lithium–sulfur batteries have emerged as a promising alternative for electrochemical energy storage, drawing significant attention for their affordability and superior theoretical capacity, approximately 1675 mAh·g^–1^, as well as a high energy density of around 2600 W·h·kg^–1^.^7−14^ In stark contrast to the operational paradigm of conventional rechargeable batteries, which employ an intercalated lithium compound as the cathode and a graphite-based structure for the anode, lithium–sulfur (Li–S) batteries adopt a fundamentally different electrochemical strategy. These batteries operate on a reversible redox reaction between sulfur and lithium sulfide (Li_2_S) at the cathode. Integral to this process is the formation and transformation of a spectrum of lithium polysulfide intermediate (Li_2_S_*n*_), with *n* signifying varying degrees of polymerization (i.e., 1, 2, 4, 6, 8). The intricate interplay of these polysulfide species not only delineates the electrochemical behavior of Li–S batteries but also critically influences their energy storage capacity, discharge efficiency, and overall cycle life. This multifaceted reaction schema, thus, emerges as a pivotal aspect in the advancement of Li–S battery technology, warranting extensive investigation to elucidate its implications on the performance and durability of these advanced energy storage systems.^2,15,16,16^

Intensive research endeavors have been undertaken to mitigate the challenges of the intrinsic insulating nature of sulfur and the dissolution of sulfur and lithium polysulfides in Li–S batteries. Among the various strategies employed, carbonaceous materials have been extensively investigated due to their superior electrical conductivity, low price, and robust mechanical attributes, aiming to encapsulate sulfur within the cathode matrix.^17−19^ Despite some efficacy in enhancing electrical contact and mechanical stability, these carbon-based hosts have exhibited limited proficiency in preventing the dissolution of Li_2_S_*n*_ intermediates and reducing the polysulfide shuttle mechanism between the anode and cathode.^20,21^ In contrast, recent advances have pivoted toward nanoengineered polar inorganic scaffolds, including various transition metal oxides and sulfides.^21,22^ These materials are recognized for their strong physicochemical affinity toward lithium polysulfides, thereby offering a promising avenue for sequestering these intermediates and attenuating the dissolution-shuttling dilemma.^13,23,24^ However, a notable trade-off is observed with these inorganic hosts, primarily concerning their inferior electrical conductivity relative to carbon substrates, which can compromise the rate capability and specific capacity of the batteries. Recent developments also point toward two-dimensional (2D) materials as well as metal-based van der Waals heterostructures as promising solutions to combat shuttling effects and improve Li–S batteries’ overall performance.^14,25−32^ Concurrently, ongoing research is focused on developing novel, cost-effective materials for sulfur hosts, aiming to enhance the commercial feasibility of Li–S batteries. This approach is crucial for overcoming material and electrochemical limitations, and advancing the field toward high-performance, durable, and cost-effective energy storage.

**Figure 1:**
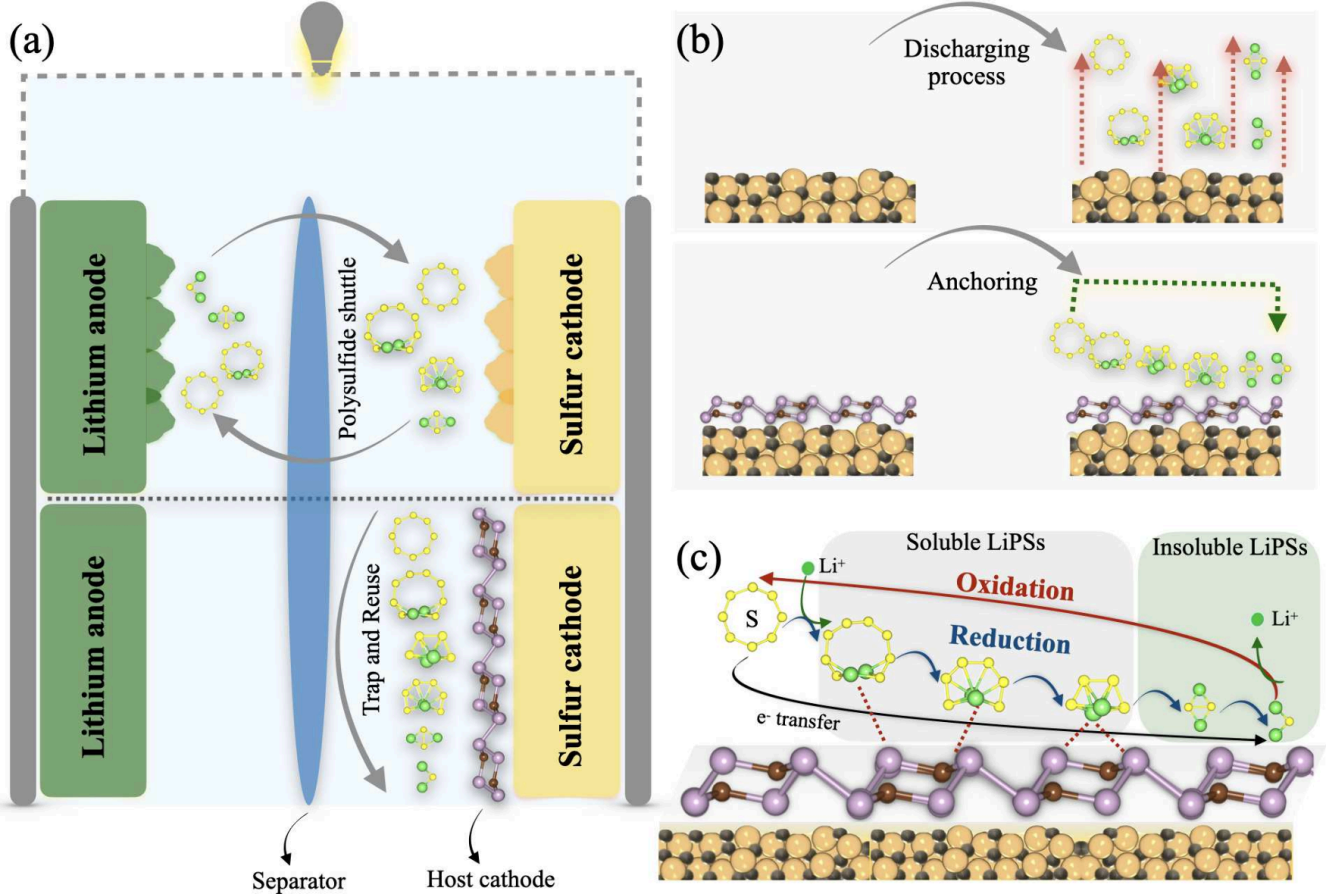
Schematic illustration of (a) shuttle effect phenomena in Li–S batteries and its suppression through (b) anchoring process and (c) catalytic effects of the 2D carbon phosphide host material.

In 1970, a family of layered materials designated as AB_3_ was successfully synthesized, including the widely studied GeP_3_ and SnP_3_.^33−35^ Subsequent computational investigations revealed the feasibility of exfoliating monolayers of GeP_3_, SnP_3_, and other group IV elements (C, Ge, Sn) from their bulk counterparts.^36−38^ These freestanding monolayers were found to exhibit robust thermodynamic and mechanical stabilities. Notably, Ramzan et al. recently reported the dynamic stability of 2D CP_3_ layered material,^39^ structurally analogous to GeP_3_ and SnP_3_. Sarkar et al. further demonstrated that CP_3_ monolayers have a low cleavage energy,^40^ facilitating their separation from the bulk phase. In this study, we designed 2D CP_3_ monolayers and examined their physical properties and structural characteristics using first-principles calculations and molecular dynamics simulations. The 2D CP_3_ monolayer, serving as an optimal S-host cathode, significantly enhanced polysulfide reaction kinetics and mitigated the shuttle effect in Li–S batteries (Figure 1a,b). Its strong binding affinity toward Li_2_S_*n*_ polysulfides (*n* = 8, 6, 4, 2, 1) effectively reduces dissolution into organic electrolytes (1,2-dimethoxyethane and 1,3-dioxolane). Moreover, the uniform adsorption of Li_2_S_*n*_ on 2D CP_3_ facilitates electrical contact, enhancing both the versatility of active materials and the conversion kinetics of Li_2_S_*n*_ polysulfides. The study also explores the interface and synergistic effects between organic electrolytes and the 2D CP_3_ monolayer, highlighting its exceptional polysulfide anchoring ability and electrical conductivity, as well as its enhanced ion diffusion and polysulfide transformation capabilities at the heterogeneous interface (Figure 1b). Consequently, the unique 2D CP_3_–S cathode demonstrates promising performance, potentially advancing research in Li–S battery technology.

## 2 Computational Frameworks

### 2.1 Density Functional Theory

Through our study, we performed first-principles calculations within the framework of Density Functional Theory (DFT) as part of the Vienna Ab Initio Simulation Package (VASP).^41^ The generalized gradient approximation in the form of the Perdew–Burke–Ernzerhof (PBE) functional^42^ was adopted self-consistently through the approach of the Projector Augmented Wave (PAW) method. The Kohn–Sham electron wave functions were expanded with an energy cutoff of 600 eV, and the convergence criteria during the structural optimizations were set to 10^–6^ eV and 10^–3^ eV/Å for energy and force, respectively. The vacuum layer during all the calculations was set to 25 Å in the *z* direction to prevent interactions between stacked layers as well as periodic images. The Monkhorst–Pack *k*-point grid of 8 × 16 × 1 was used in the reciprocal space during the geometrical optimizations.^43^ The charge transfer between atoms was evaluated based on the Bader charge analysis algorithm.^44^

### 2.2 Classical Molecular Dynamics Simulations

We considered electrolyte solutions that consist of Li_2_S_6_ and Li_2_S_8_, which are diluted with either DOL or DME. The OPLS-AA force field was used to build up the interaction potential models of the solvents and CP_3_ monolayer.^45,46^ The CL&P force field for ionic liquids^47^ is used to model the interaction potential for ions with the corrected parameters proposed by Rajput et al.^48^ All MD simulations were conducted using the LAMMPS software package,^49^ the three-dimensional periodic boundary conditions are applied, and a time-step δ*t* = 1 fs is used for all simulated cases. The initial distribution was generated using the Moltemplate package,^50^ where the simulated system constitutions are placed randomly into a monoclinic box. Subsequently, the system was equilibrated in multiple stages to ensure stability and accuracy. The system is agitated using the Langevin thermostat at *T* = 500 K for one ns, followed by the same process using the Nosé–Hoover thermostat.^49^ The simulated systems were equilibrated in the *NPT*-statistical ensemble using the Berendsen barostat to reach the desired ambient condition of temperature and pressure (*T* = 303 K, *P* = 1 bar).^49^

In the first stage, *NPT* MD simulations were performed from *T* = 500 K to *T* = 303 K under constant pressure *P* = 500 bar to achieve the desired temperature over *t* = 1 ns. Next, the pressure was adjusted from *P* = 500 bar to *P* = 1 bar at a constant temperature of *T* = 303 K, facilitating volume stabilization over another *t* = 1 ns. In the last performed *NPT* equilibration stage, the systems were equilibrated at room temperature and ambient pressure (*T* = 303 K, *P* = 1 bar) for *t* = 10 ns. Finally, we run the simulations for a long time up to *t* = 100 ns to produce the static and dynamic properties in the *NVT* statistical ensemble using the Nosé–Hoover thermostat at *T* = 303 K and fixed volume. We note that the equilibrated box size depends on the simulated system, characterized by a salt type and solvent type and amount. However, the base area delimited by the CP_3_ size, a parallelogram of length *L*_*x*_ = 37.09 Å and width *L*_*y*_ = 32.12 Å with a tilt factor xy = −18.5445 Å, remains fixed for all simulated cases.

The structural analysis was conducted using the radial distribution function (RDF), *g*(*r*), and coordination number, *N*_c_.^51,52^ The RDF, *g*(*r*), quantifies the variation of particle density with distance from a reference particle, providing a measure of the local structure.
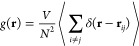
1where *N* is the number of particles and *V* is the volume of the simulated system. The first peak in *g*(*r*) represents the nearest neighbors, revealing the most probable interparticle distance and interaction strength through the relation *g*(*r*) = e^–β*U*(*r*)^, where  and *U*(*r*) is the interaction potential.^53^ Subsequent peaks correspond to higher-order neighbors, providing insights into medium-range order, coordination numbers, and packing efficiency. In isotropic, homogeneous systems, *g*(*r*) approaches 1 at large distances, indicating a uniform particle distribution.

The coordination number *N*_c_ is defined as the number of neighbor particles that are within the specified cutoff distance of the pair interaction potential from a central particle:
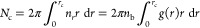
2where *r*_c_ is the position at which the interaction potential between particles goes to zero, *n*_b_ is the bulk density, and *n*_*r*_ is the average number density of the considered particles at a given distance *r*, related to the RDF by *n*_*r*_ = *n*_b_*g*(*r*).

Thus, the coordination number at a distance *r*, *N*(*r*), is defined by
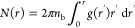
3where *r* is an arbitrary distance from a tagged particle.

The ionic conductivity σ is calculated using the collective mean-square displacement:^54^
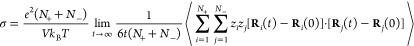
4where *N* is the number of ions, *V* is the volume of the simulated system, *e* is the elementary charge, *k*_B_ is Boltzmann’s constant, *T* is the temperature, *z*_*i*_ is the ion charge, and **R**(*t*) and **R**(0) are the center-of-mass position vectors of an ion at times *t* and 0, respectively. The cross terms (*i* ≠ *j*) in this equation account for the correlation of different ion displacements.

The Nernst–Einstein ionic conductivity σ_ne_ is calculated using the self-diffusion of ions. It can be an acceptable assessment of the real ionic conductivity if ionic species motion is uncorrelated:
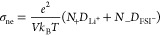
5where *N*_+_ and *N*_–_ are, respectively, the number of cations and anions, *e* is the elementary charge, *D*_Li^+^_ and *D*_FSI^–^_ are the self-diffusion coefficients of Li^+^ and FSI^–^, *V* is the volume, *k*_B_ is Boltzmann’s constant, and *T* is the temperature.

The cation transference number is assessed by
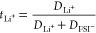
6where the self-diffusion coefficients are determined from the mean-square-displacement, calculated from the MD simulations as follows:
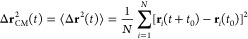
7In the expression above, **r**_*i*_(*t*) represents the temporal position of a random walker *i* (an anion or a cation), *t* indicates the time, and *t*_0_ is the initial time at which the random walker begins to move.

The collective diffusion coefficient and self-diffusion coefficients are extrapolated from the normal diffusion regime:
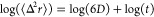
8

## 3 Results and Discussion

The electrochemical reactions on 2D CP_3_ S-host cathode of Li–S batteries are illustrated in Figure 1c. The discharge sequence initiates with the adsorption of S_8_ molecules onto the catalyst’s surface, sparking the sulfur reduction reaction (SRR). This leads to the formation of long-chain, soluble lithium polysulfides (LiPSs) such as Li_2_S_8_, Li_2_S_6_, and Li_2_S_4_. A crucial aspect of this phase is mitigating the shuttle effect by ensuring that the 2D CP_3_ catalyst has a strong affinity for these LiPSs to prevent their dissolution in the electrolyte, thereby stabilizing them. As discharge continues, the reaction culminates in the formation of insoluble LiPSs, specifically Li_2_S_2_ and Li_2_S, with the process being expedited by effective catalysts. During charging, Li_2_S is oxidized back to S_8_, with the catalyst playing a pivotal role in lowering the energy barrier for Li_2_S decomposition. Additionally, the catalyst’s metallic nature is vital for facilitating rapid electron transfer throughout the reaction. Optimal catalysts for Li–S batteries are thus characterized by their metallic nature, moderate adsorption of soluble LiPSs, fast reaction kinetics of SRR, and low Li_2_S decomposition barrier.

### 3.1 Anchoring Ability of 2D CP_3_ toward LiPSs

**Figure 2:**
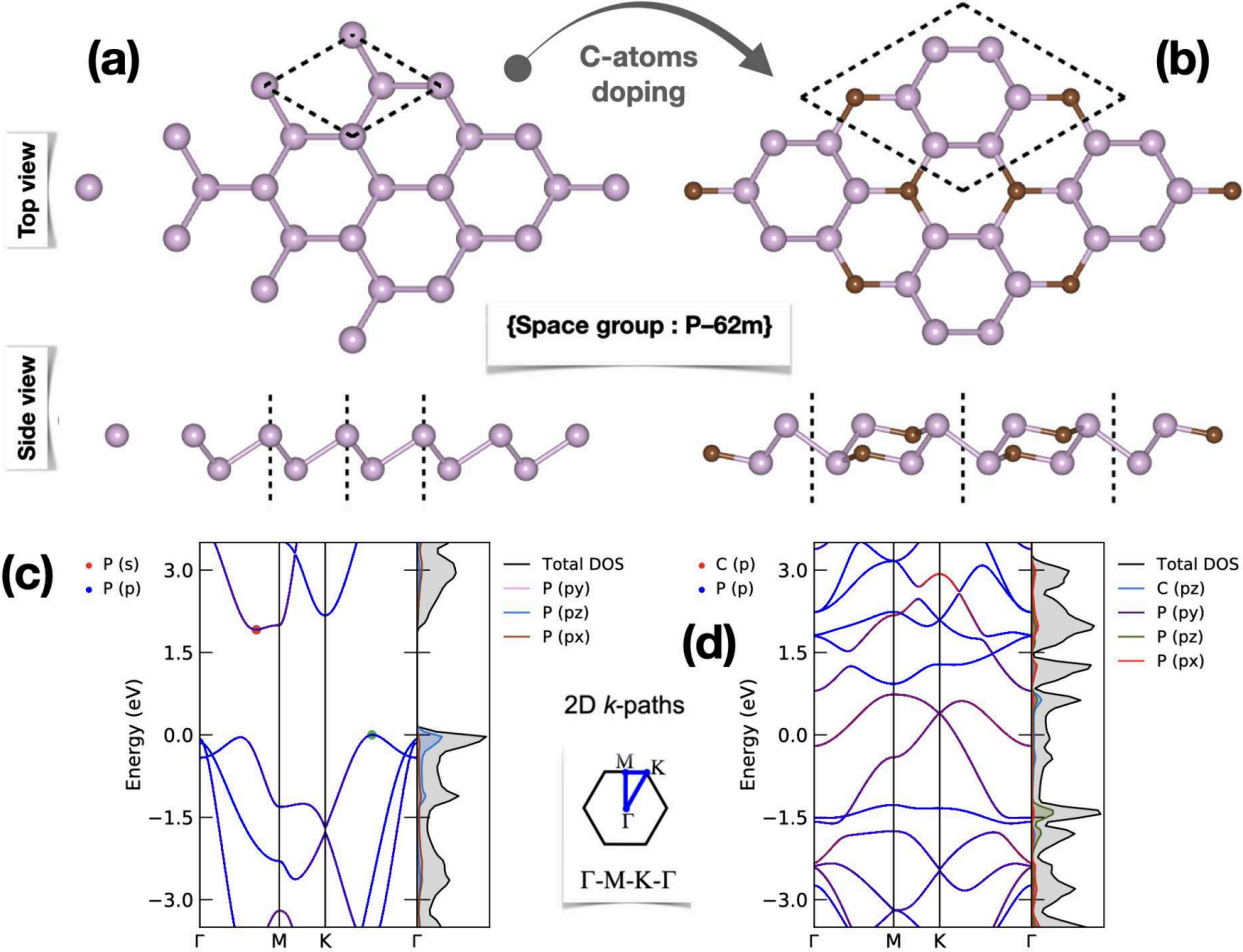
(a, b) Top and side views of free-standing (a) blue phosphorus and (b) CP_3_ monolayers. (c, d) Projected band structures of the (c) blue phosphorus and (d) CP_3_ monolayers computed using GGA-PBE with the corresponding projected densities of states.

Recently, Ramzan et al. introduced the 2D CP_3_ monolayer via first-principle calculations as a new entrant in the carbon phosphide family.^39^ Characterized by its distinctive puckered crystalline structure, similar to SnP_3_ and GeP_3_, the CP_3_ monolayer is a candidate for experimental exfoliation from its layered bulk form or by integrating carbon atoms into blue phosphorene. As illustrated in Figure 2a,b, the primitive cell of CP_3_ comprises eight atoms forming alternating C–P and P–P bonds. The Bravais lattice vectors are obtained as *a* = *b* = 6.218 Å, with bond lengths of 1.783 Å for C–P and 2.279 Å for P–P, consistent with previously reported findings.^39,40,55^ The computed electronic band structure and the associated projected density of states for both Blue phosphorus and CP_3_ monolayers are depicted in Figure 2c,d. The findings indicate that CP_3_ monolayer exhibits a distinctly metallic electronic structure compared to the blue phosphorene which shows a semiconductor nature, suggesting that 2D CP_3_ monolayer has promising conductivity properties for use as active electrode materials. Additionally, the projected density of states reveals that the electronic states close to the Fermi level are predominantly composed of the p-orbitals from both carbon and phosphorus atoms.

An expanded supercell structure, specifically a 3 × 3 × 1 configuration of the 2D CP_3_ monolayer, was employed to examine the binding efficacy of S_8_ and Li_2_S_*n*_ polysulfides (*n* = 8, 6, 4, 2, 1). This study involved a comprehensive optimization of multiple placements of S_8_ and LiPS clusters across different active sites on the CP_3_ surface to determine the most energetically favorable binding configurations. The stability of these configurations was assessed by calculating the binding energy (*E*_b_) using the following equation, evaluated both with and without van der Waals (vdW) corrections:
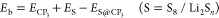
9where *E*_S@CP_3__ and *E*_CP_3__ represent the total energies of the CP_3_ surface after and before adsorption, respectively, while *E*_S_ corresponds to the total energy of S_8_ or Li_2_S_*n*_ in its bulk reference state. In this study, the binding energy is defined as positive for exothermic interactions, indicating energy release during adsorption and the stabilization of S_8_/Li_2_S_*n*_ molecules on the CP_3_ surface. This sign convention aligns with the equation provided and ensures consistency with the interpretation of adsorption stability.

The inclusion of vdW corrections significantly impacts the calculated binding energies, emphasizing the importance of dispersion interactions in accurately capturing the adsorption process. Incorporating vdW contributions ensures a more realistic description of the weak interfacial forces between polysulfides and the CP_3_ surface, which would otherwise be underestimated in noncorrected calculations. The calculated positive *E*_b_ values indicate stable adsorption, promoting a uniform distribution of S_8_/Li_2_S_*n*_ molecules across the CP_3_ surface. This uniformity is critical for preventing clustering and dendrite formation during battery cycling, thereby enhancing electrode kinetics and the long-term stability of the system. Such properties are essential for improving the overall electrochemical performance of lithium–sulfur batteries, ensuring controlled and efficient energy storage processes.

**Figure 3:**
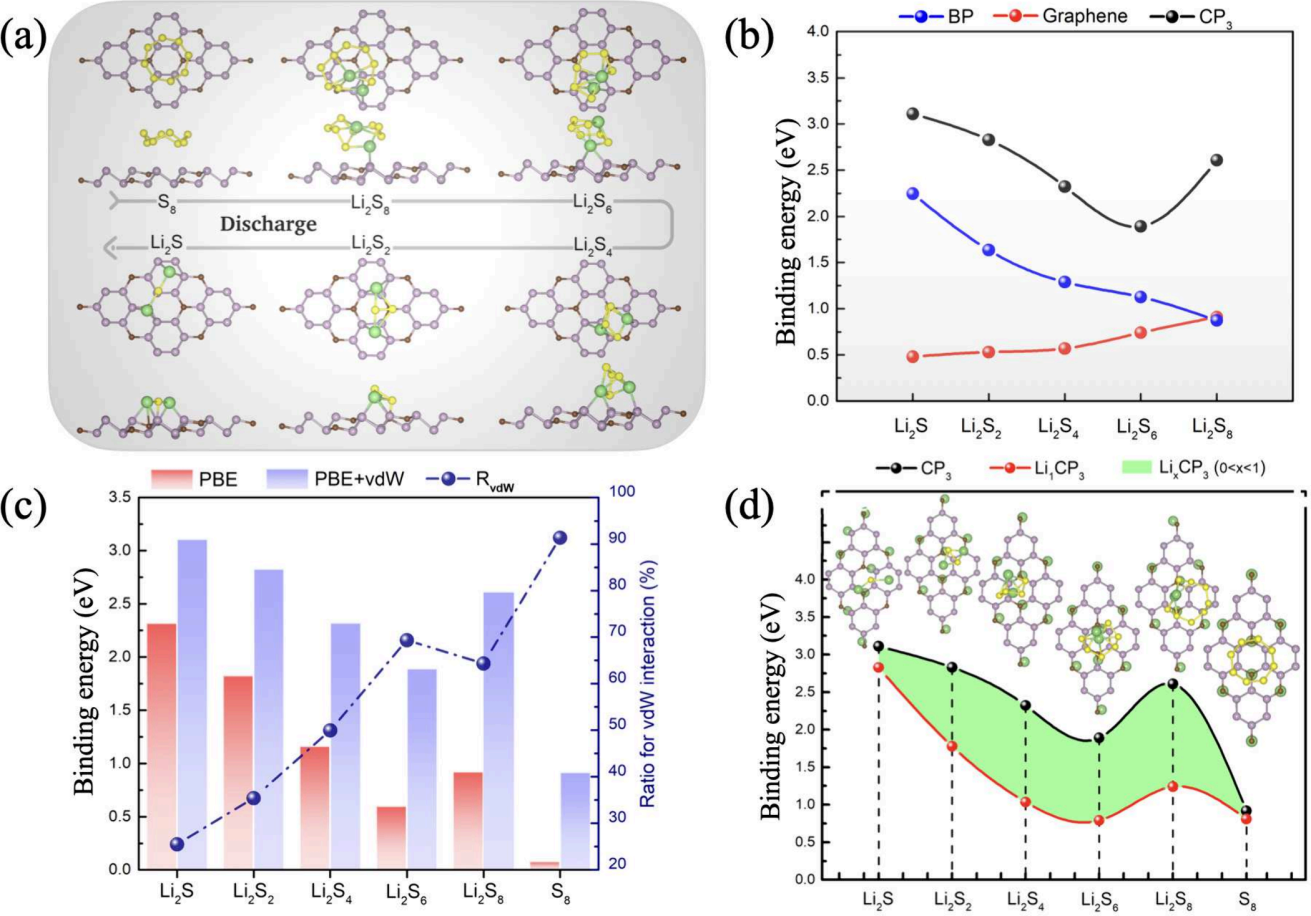
(a) The top and side views of the fully optimized structures of S_8_/Li_2_S_*n*_ molecules on CP_3_ monolayer. (b) Computed binding energies of Li_2_S_*n*_ molecules on blue phosphorene, graphene, and CP_3_ monolayers. (c) Computed binding strengths of S_8_/Li_2_S_*n*_ molecules on CP_3_ monolayer through DFT and DFT-D3 correction and their corresponding ratio (*R*_vdW_). (d) Computed binding energy with different Li-concentrations on the CP_3_ monolayer.

The configurations exhibiting the highest binding energy, indicative of the highest stability, are presented across the explored binding sites and orientations in Figure 3a. The affinities of S_8_ and Li_2_S_*n*_ molecules on the CP_3_ surface are summarized in Table 1. Our computation reveals that the S_8_ cluster attaches primarily above the C_2_P_4_-hexagon rings, maintaining a parallel orientation to the CP_3_ surface with a minimum binding distance of approximately 3.421 Å, significantly exceeding the lengths of P–P and C–P bonds. The adsorption energy of an S_8_ molecule on the CP_3_ layer is obtained to be 0.917 eV when vdW corrections are considered. This suggests that the adsorption is primarily governed by vdW forces, coupled with a minimal charge transfer of around −0.003|*e*| from the CP_3_ layer to the S_8_ cluster, indicating a minor redistribution of electrons.

**Table 1:** Computed Binding Energies of S_8_/Li_2_S_*n*_ Molecules on the CP_3_ Surface through DFT and DFT-D2 Correction (*E*_b_^DFT^ and *E*_b_^DFT-D2^), Charge Transfer Δ*Q* (|*e*|) between S_8_/Li_2_S_*n*_ and the Anchoring Material, and Shortest Binding Height (*h*)

molecule	*E*_b_^DFT^ (eV)	*E*_b_^DFT-D2^ (eV)	Δ*Q* (|*e*|)	*h* (Å)
S_8_	0.079	0.917	–0.003^a^	3.421
Li_2_S_8_	0.922	2.608	0.291	2.266
Li_2_S_6_	0.599	1.891	0.371	2.108
Li_2_S_4_	1.162	2.322	0.537	1.838
Li_2_S_2_	1.826	2.828	0.662	1.772
Li_2_S	2.317	3.109	0.802	1.428

a Δ*Q* < 0 reveals electron transfer from the host CP_3_ monolayer to S_8_.

In a comparative analysis, the binding strengths of S_8_/Li_2_S_*n*_ molecules on CP_3_ surfaces were juxtaposed against those on blue phosphorene and graphene monolayers. As illustrated in Figure 3b, the binding energy trends observed on the CP_3_ surface display a nonlinear relationship, initially decreasing from Li_2_S to Li_2_S_6_ and then incrementing for longer polysulfide chains. In contrast, blue phosphorene exhibited a monotonically decreasing trend in binding energy with increasing chain length of Li_2_S_*n*_, indicating progressively weaker interactions with larger polysulfide molecules. Conversely, the graphene monolayer consistently demonstrated the lowest binding energies across the spectrum of chain lengths, signifying the least robust interaction with the Li_2_S_*n*_ molecules. These distinct binding profiles highlight the potential of CP_3_ in fostering stronger interactions with lithium polysulfide chains. This characteristic may prove advantageous in the realm of Li–S battery technology, particularly in enhancing the stability and efficiency of electrode materials through more robust binding affinities.

To elucidate the specific anchoring mechanisms and assess the relative significance of chemisorption versus vdW physisorption, an analysis has been conducted calculating the proportion of vdW contributions for various sulfur-containing molecules. This ratio is defined as
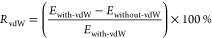
10where *E*_with-vdW_ represents the binding energy including vdW interaction, and *E*_without-vdW_ denotes the binding energy excluding vdW interaction including vdW interaction. As demonstrated in Figure 3c, vdW interactions predominate in the nonlithiated state, with an *R*_vdW_ value of approximately 91.36%, suggesting that the S_8_ molecule predominantly adheres to the CP_3_ monolayer through vdW forces rather than chemical bonding. Throughout lithiation, the contribution of vdW interactions to the total binding energy of Li_2_S_*n*_ (*n* = 8, 6, 4) remains considerable, with ratios ranging from 68.32% to 49.95%. As lithiation intensifies, the prevalence of physical interactions diminishes notably for Li_2_S_2_ and Li_2_S, with the *R*_vdW_ ratio falling between 35.42% and 25.49%. The changing adsorption energies of S_8_/Li_2_S_*n*_ molecules (*E*_b_) underscore the influential role of Li atoms in chemical bonding and the predominance of S atoms in vdW interactions. Notably, the smallest binding energy is observed for S_8_. Conversely, the CP_3_ monolayer demonstrates substantial large binding energies toward Li_2_S_*n*_ molecules, indicating a balanced interaction profile. Similar trends were observed by varying the Li concentration on the CP_3_ monolayer as illustrated in Figure 3d.

**Figure 4:**
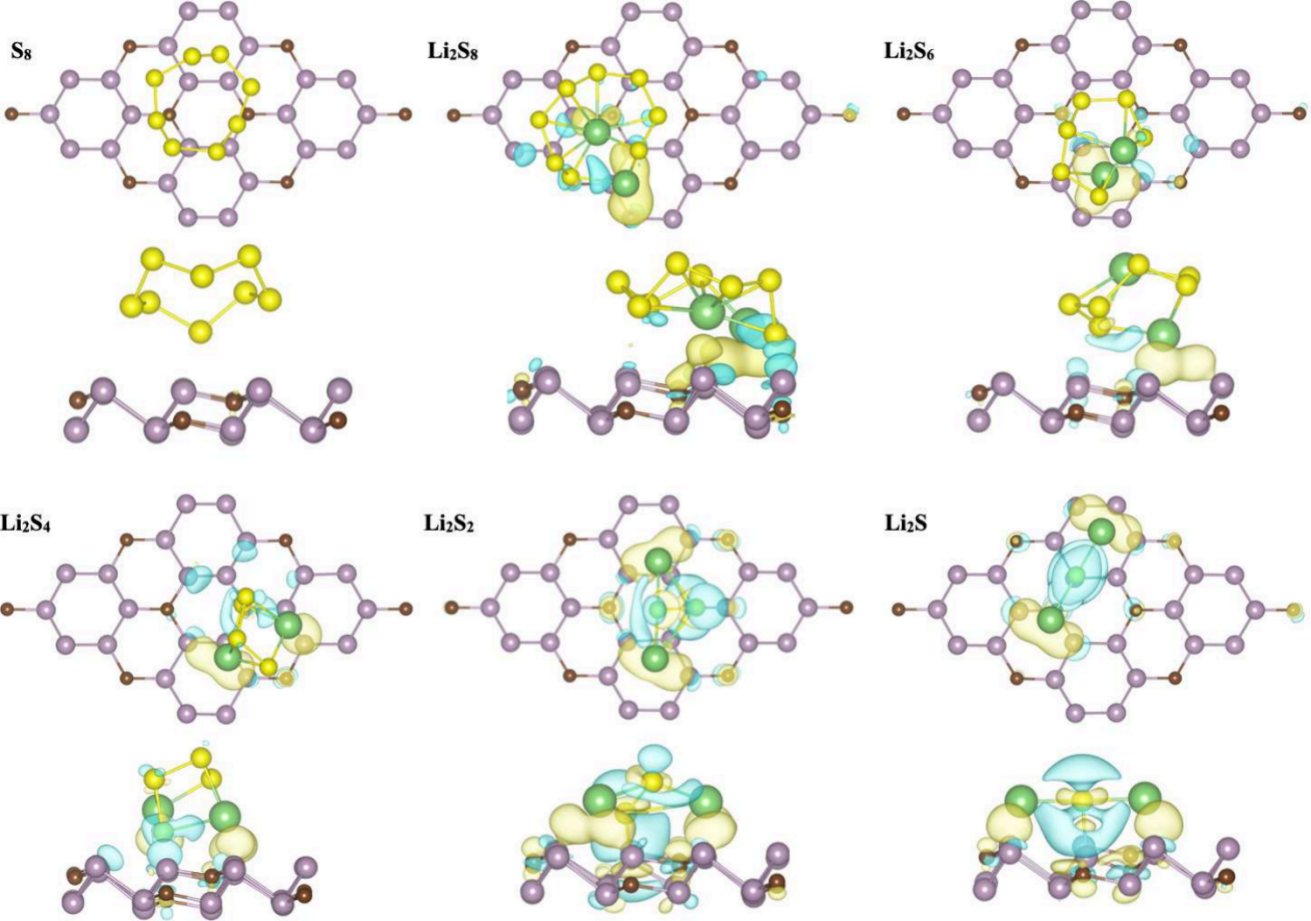
Distributed charge density for S_8_/Li_2_S_*n*_ molecules adsorbed on the CP_3_ monolayer. Yellow and blue colors indicate the electron accumulation and depletion, respectively. The value of the isosurface is set to be 0.001 e Å^–3^.

To elucidate the binding dynamics and chemical bond formation following the adsorption of S_8_/Li_2_S_*n*_ molecules, an in-depth investigation was undertaken into the electronic structure alterations, specifically analyzing the charge density difference (*Δρ*) of S_8_/Li_2_S_*n*_ clusters adsorbed onto the CP_3_ monolayer by employing the following shortest binding height:
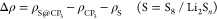
11where ρ_CP_3__ refers to the electron charge density of the pristine CP_3_ monolayer, ρ_S@CP_3__ denotes the charge density of the composite S_8_/Li_2_S_*n*_@CP_3_ system, and ρ_S_ represents the charge density of the isolated S_8_ and Li_2_S_*n*_ clusters, all maintained under identical structural parameters and without geometrical relaxation. Illustrated through 3D isosurface charge density figures, as depicted in Figure 4, the alterations in charge distribution reveal regions of charge accumulation and depletion. The computed charge redistribution primarily occurs via Li–S and P–S bonding, particularly in the Li_2_S_2_ and Li_2_S molecules, with a notable charge near the P atoms and Li ions. As the discharge process happens from Li_2_S_8_ to Li_2_S, an increase in charge transfer correlates with the changing sulfur concentration, a trend that complements the computed variation in the binding strength. This correlation underscores the importance of such detailed electronic studies in predicting and optimizing material performance and stability in practical applications.

### 3.2 Gibbs Free Energies Associated with the SRR

**Figure 5:**
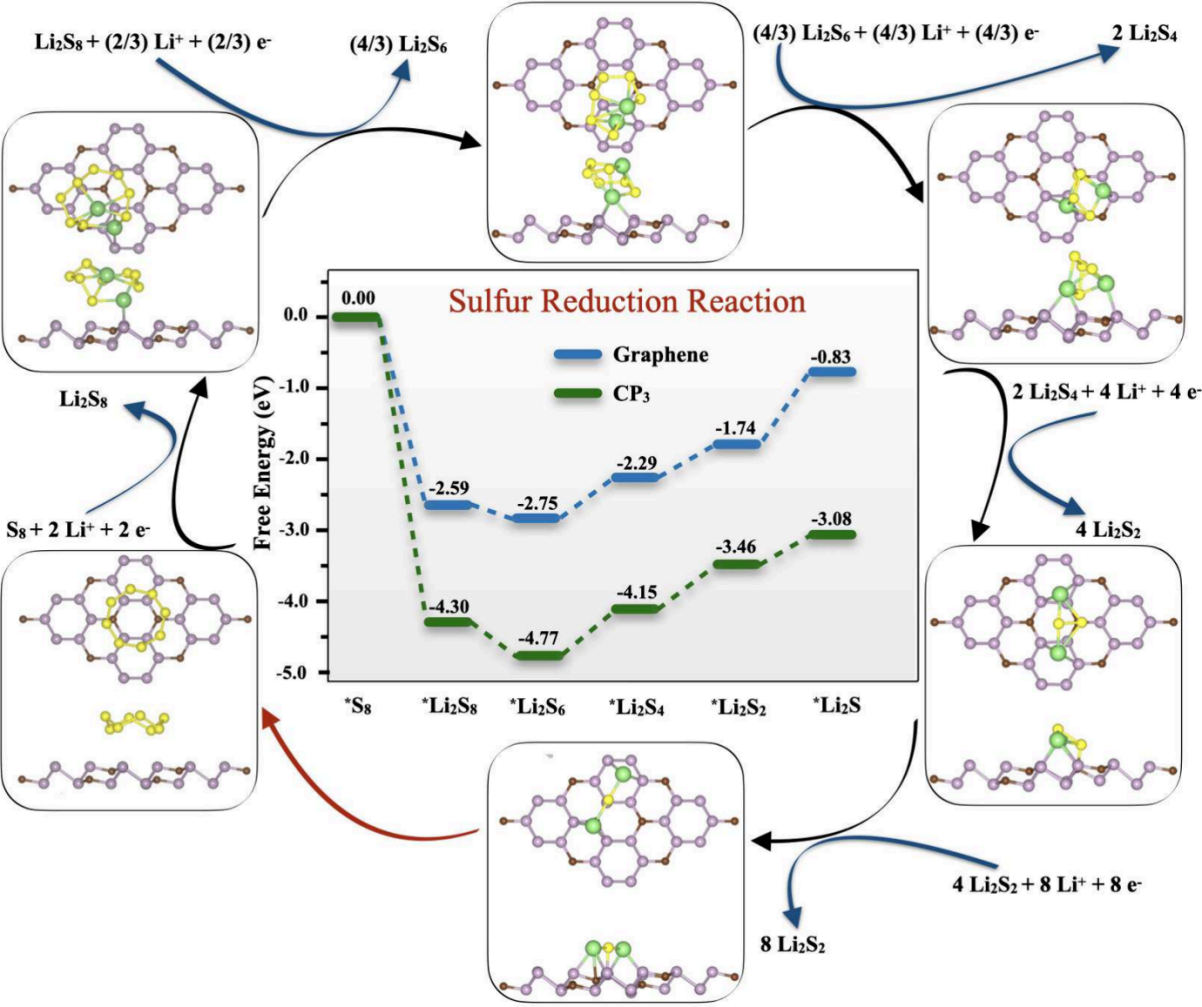
Free energy landscape of the sulfur reduction reaction on graphene and CP_3_ surfaces, with insets highlighting the optimized geometries of reaction intermediates.

The CP_3_ surface has demonstrated substantial enhancement in the redox kinetics of sulfur species as an electrocatalyst, thereby significantly boosting the performance of Li–S batteries. Compared to graphene and blue phosphorene, the CP_3_ catalyst has been shown to decrease the activation energies required for the SRR. This improvement is attributed to the effective adsorption of sulfur species onto the CP_3_ surface, facilitated by phosphorus–lithium (P–Li) bond formation. This bonding interaction contributes to the weakening of sulfur–sulfur (S–S) bonds, which in turn accelerates their dissociation, thus promoting faster reaction kinetics. The enhanced kinetics of SRR on the CP_3_ monolayer is supported by the Gibbs free energy diagram depicted in Figure 5. The diagram compares the Gibbs free energies of the SRR pathway on CP_3_ and graphene surfaces. For the initial reduction steps from S_8_ to Li_2_S_8_, and subsequently to Li_2_S_6_, both CP_3_ and graphene exhibit negative Gibbs free energy changes, indicative of exothermic and spontaneous reaction sequences. The more pronounced negative energy change on the CP_3_ surface, compared to graphene, underscores a thermodynamically more favorable reduction process.

However, the later stages of reduction leading to the formation of solid Li_2_S_4_, Li_2_S_2_, and Li_2_S are characterized by positive Gibbs free energy changes on both CP_3_ and graphene, suggesting endothermic and nonspontaneous reactions. Notably, CP_3_ significantly lowers the Gibbs free energy pathway in comparison to graphene, indicative of a lower energy pathway for the reaction to proceed. The clear evidence from the free energy profiles points to a comprehensive acceleration of the SRR kinetics when mediated by the CP_3_ surface. This is primarily due to the excellent catalytic properties of CP_3_, which facilitate more energetically favorable pathways for the successive reduction steps in the SRR, as opposed to the pathways available on graphene. Consequently, the CP_3_ catalyst emerges as a more potent material for improving the efficiency and lifetime of Li–S batteries.

### 3.3 Catalytic Decomposition of Li_2_S_*n*_ on CP_3_

Additionally, we delved into the decomposition barrier of the discharge end product in Li–S batteries. The charging phase is often hindered by the low electronic conductivity and the substantial energy barrier required to decompose the final discharge product, Li_2_S, leading to increased cell overpotential and impacting the rate capability of the batteries. The electrochemical efficiency of Li–S batteries is significantly influenced by the kinetics of Li_2_S decomposition, which involves breaking of Li–S bonds, and the diffusion of Li^+^ ions on the 2D CP_3_ substrate. For this analysis, Climbing Image Nudged Elastic Band (CI-NEB) calculations were employed to assess the energy barrier in the reaction Li_2_S → LiS + Li^+^ + e^–^. This reaction entails the energy necessary to cut off Li–S bonds at the most favorable adsorption site and facilitate the migration of Li^+^ to another stable site. The energy profiles and reaction pathways for this process are detailed in Figure S1a,b.

Our computed results reveal a relatively low Li diffusion barrier of 0.18 eV for Li-ion on CP_3_ surface, consistent with findings from previous research.^55^ Prior experimental investigations have shown that a high decomposition energy barrier adversely impacts the cell voltage, indicating that minimizing this barrier is crucial for optimal Li–S battery performance. A substantial large adsorption energy of Li_2_S on the substrate is expected to correlate with the decomposition energy barrier. In contrast, isolated gas-phase Li_2_S has previously been shown to have a large decomposition energy barrier of 3.59 eV.^56−58^ Notably, the CP_3_ substrate seems to effectively lower this barrier to 0.95 eV as depicted in Figure S1b, a significant reduction compared to other 2D materials, such as BAs and B_2_N_2_.^14,59^ However, we note that the decomposition barriers for BAs and B_2_N_2_ were computed using the DFT-D3 method for van der Waals corrections, whereas our study employed the DFT-D2 method. While these methods differ in their parametrization for dispersion effects, both are consistent in capturing trends in decomposition behavior. Consequently, our comparison is qualitative, aimed at highlighting general trends rather than providing exact quantitative values. This distinction underscores the efficacy of the CP_3_ substrate in lowering the decomposition barrier compared to other 2D materials, independent of the methods employed. The combination of strong adsorption energy and reduced decomposition barrier suggests that the CP_3_ substrate could enhance the oxidative decomposition of Li–S bonds, thereby improving the electrode kinetics in Li–S batteries.

### 3.4 Solvation Structure and Dynamics of Li_2_S_6_/Li_2_S_8_-based electrolytes in the presence of CP_3_ surface

Studying Li_2_S_6_/Li_2_S_8_ structures and dynamics in our proposed cathode model for Li–S batteries is crucial due to their roles in the electrochemical reactions during charging/discharging cycles. These intermediate polysulfides, which are formed during the reduction and oxidation of sulfur, contribute to a high energy density. All current in the atomistic mechanisms are produced through the Li_2_S_8_ reduction until this species is consumed.^60^ The formation of Li_2_S_6_ and Li_2_S_8_ is associated not only with the shuttle effect but also with low ionic conductivity and limited solubility in ether-based liquid electrolytes.^61,62^ Thus, understanding their behavior near the CP_3_ monolayer in the presence of solvent using classical MD simulation is crucial for making the current approach more robust. the simulated systems are composed of a CP_3_ monolayer, containing 216 P atoms and 72 C atoms, in contact with 27 (Li_2_S_6_/Li_2_S_8_) salt molecules, in the presence of a solvent, dimethoxyethane (DME) or diethylene glycol dimethyl ether (DOL). The solvent is added in three different amounts, where the molar fractions *n*(solvent)/*n*(salt) = 0.3, 1, and 3.

### Solvation Structure

**Figure 6:**
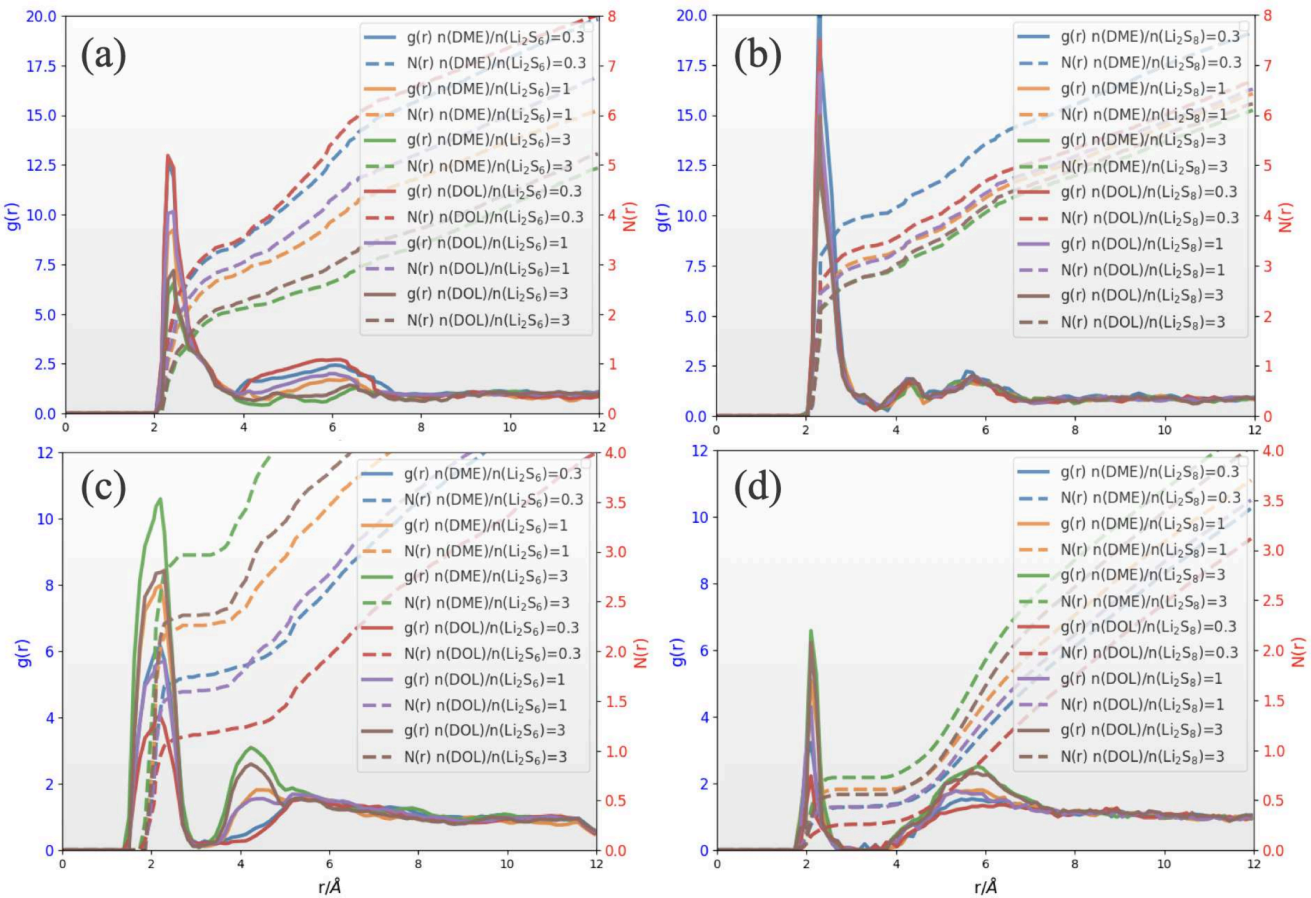
Comparison of Radial Distribution Function (*g*(*r*)) and Coordination Number (*N*(*r*)) average profiles for S_6_^2–^ and S_8_^2–^ anions (S^δ−^, where δ denotes the partial charge on each S atom) surrounding a single Li^+^ ion in simulated electrolytes near the CP_3_ surface, with varying solvent amounts. (a, b) depict Li_2_S_6_/DME and Li_2_S_6_/DOL electrolytes, as well as Li_2_S_8_/DME and Li_2_S_8_/DOL electrolytes, respectively. (c, d) illustrate the average profiles of solvent oxygen atoms around a single Li^+^ ion in the same electrolytes.

The MD simulations performed within this work focus on examining the solvation structure and diffusion of Li^+^ ions in Li_2_S_6_/DME, Li_2_S_6_/DOL, Li_2_S_8_/DME and Li_2_S_8_/DOL electrolytes. To investigate the solvation structure of Li^+^ in Li_2_S_6_ and Li_2_S_8_-based electrolytes, we analyzed the time-averaged radial distribution function (*g*(*r*)) over the last 50 ns of the MD simulation, with a sampling interval of 100 ps. We present the average profile of the *g*(*r*) between Li^+^ and sulfur ions in Figure 6a,b, and the *g*(*r*) between Li^+^ and solvent molecules (DME or DOL) in Figure 6c,d, for varying solvent amounts, *n*(solvent)/*n*(salt) = 0.3, 1, and 3. The first peaks in the radial distribution function *g*(*r*) of sulfur around Li^+^ are observed at approximately 2.32 Å for both Li_2_S_6_- and Li_2_S_8_-based electrolytes, regardless of the type and amount of added solvent, as shown in Figure 6a,b. These peaks are more pronounced in the Li_2_S_8_ electrolytes due to their higher sulfur content compared to the Li_2_S_6_ electrolytes. It is important to note that the coordination between Li^+^ ions and S atoms decreases with increasing solvent amount (DME or DOL) for both Li_2_S_6_- and Li_2_S_8_-based electrolytes because the coordination of Li^+^ with solvent molecules increases. According to Figure 6c,d, Li^+^ ions are solvated by the solvent molecules, with the most probable distance between solvent and Li^+^ ion being approximately 2.13 Å. Furthermore, the Li^+^ cations in the Li_2_S_6_-based electrolyte (Figure 6c) are more solvated than those in the Li_2_S_8_-based electrolyte (Figure 6d), as evidenced by the higher peaks in the *g*(*r*) curves of Li^+^–solvent in the Li_2_S_6_ electrolyte and the corresponding coordination number values. Thus, solvents are closer to the Li^+^ ions than to the sulfur ions, and they play a crucial role in the dissociation of ions. In Figures S2 and S3, we present the statistical distribution of Li^+^ coordination number values obtained from solvents, sulfur ions, and its surrounding Li^+^. Note that threshold distances are used to calculate the coordination number of the first shell around a tagged Li^+^. The threshold distance for solvent and sulfur ions is 3 Å, while for its surrounding Li^+^ ions it is 5 Å. From Figures 6c,d, it can be observed that the height of the *g*(*r*) peaks indicates an increase in the interaction of Li^+^ with solvents as the amount of solvent increases, thereby affecting the coordination of Li^+^ ions. Accordingly, as depicted in Figures S2 and S3, the most probable coordination numbers (*N*(*r*)) of a tagged Li^+^ from its surrounding Li^+^ ions are ≤2 for *n*(DME)/*n*(Li_2_S_6_) = 3, 1 ≤ *N*(*r*) ≤ 3 for *n*(DOL)/*n*(Li_2_S_6_) = 3, 1 ≤ *N*(*r*) ≤ 4 for *n*(DME)/*n*(Li_2_S_8_) = 3, and 2 ≤ *N*(*r*) ≤ 5 for *n*(DOL)/*n*(Li_2_S_8_) = 3. The most probable coordination numbers of Li^+^ with sulfur ions are 1 ≤ *N*(*r*) ≤ 4 for *n*(DME)/*n*(Li_2_S_6_) = 3, 2 ≤ *N*(*r*) ≤ 6 for *n*(DOL)/*n*(Li_2_S_6_) = 3, 4 ≤ *N*(*r*) ≤ 7 for *n*(DME)/*n*(Li_2_S_8_) = 3, and 5 ≤ *N*(*r*) ≤ 7 for *n*(DOL)/*n*(Li_2_S_8_) = 3. Accordingly, DME dissociates Li_2_S_8_ and Li_2_S_6_ more effectively than DOL does. Furthermore, DME is more effective than DOL in solvating Li^+^ cations. Thus, the presence of a solvent facilitates favorable coordination between Li^+^ and S during chemical reactions to form Li_2_S_2_ and Li_2_S. Figure S4 illustrates the *g*(*r*) and *N*(*r*) between Li^+^–P and P–S^δ−^. The flat *g*(*r*) curves, i.e., absence of sharp peaks in the *g*(*r*) curves, in Figure S4, indicate a uniform distribution of Li^+^ and sulfur ions around the CP_3_ monolayer. The absence of sharp peaks in the *g*(*r*) between P–S^δ−^ and Li^+^–P curves indicates a moderate interaction between P–S^δ−^ and Li^+^–P. This interaction decreases with increasing solvent amount and also suggests that there are no preferred distances or specific clustering patterns of ions in proximity to the CP_3_ monolayer. This information can be used to develop strategies that guide the deposition process and ensure the ions’ normal diffusion.

### Transport Properties

**Figure 7:**
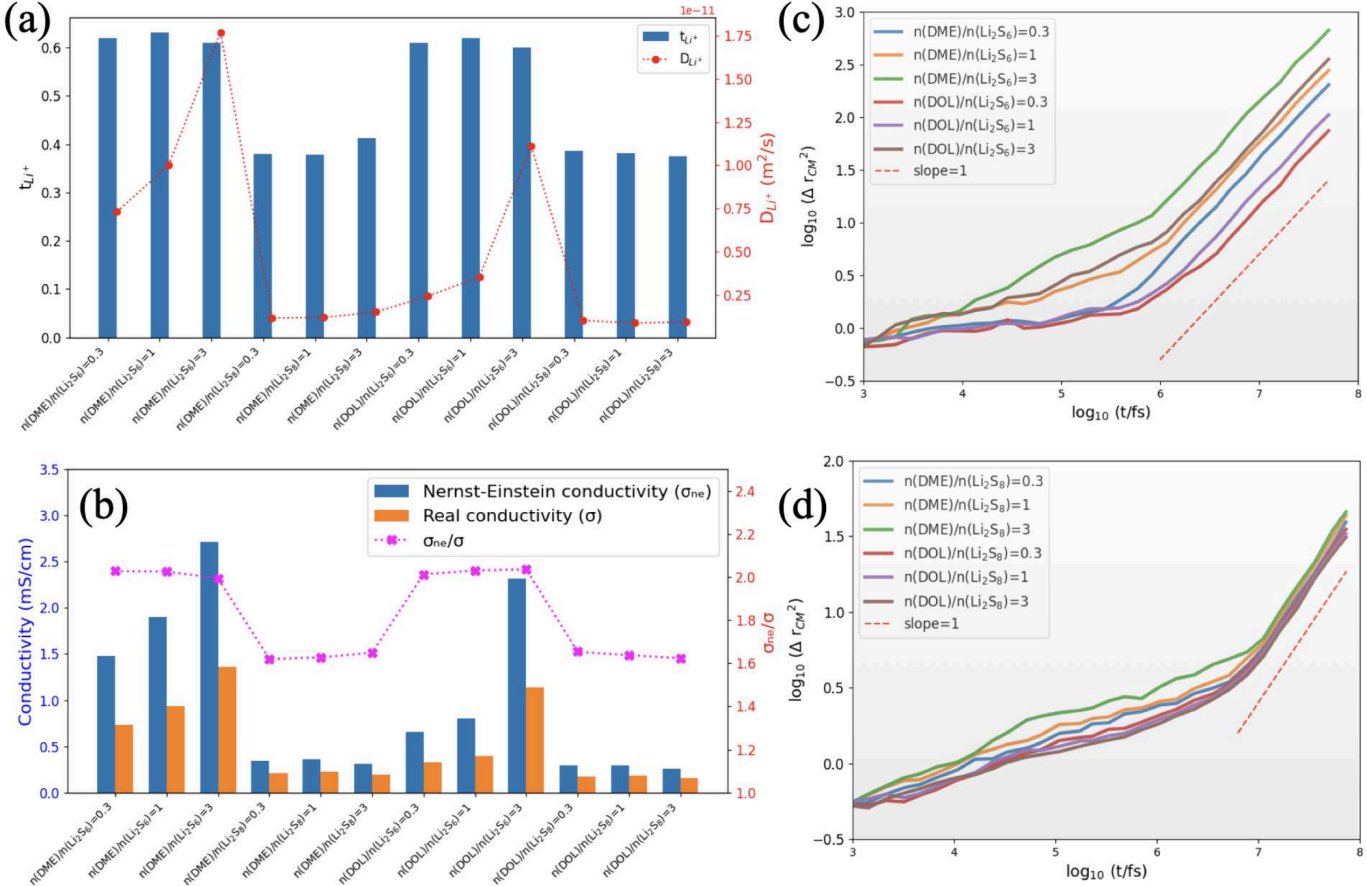
Relevant dynamics properties for the simulated electrolyte near the CP_3_ monolayer: (a) Li^+^ transference number (*t*_Li^+^_) and the Li^+^ self-diffusion coefficient, calculated for Li_2_S_6_/DME, Li_2_S_6_/DOL, Li_2_S_8_/DME and Li_2_S_8_/DOL simulated systems. (b) Ionic conductivity values (σ) and Nernst–Einstein conductivity (σ_ne_) and its comparison. The dotted lines serve as visual guides. (c) and (d) Log–Log plot of the Li^+^ MSD variation as a function of time. (c) Li_2_S_6_/DME and Li_2_S_6_/DOL, (d) Li_2_S_8_/DME and Li_2_S_8_/DOL. The MSD plots confirm that a normal diffusion mode is achieved, which confirms the quality of the calculated dynamic properties.

As depicted in Figure 7a–d, we observed a significant difference in the diffusion behavior between the Li_2_S_6_ and Li_2_S_8_ electrolytes. The diffusion of Li^+^ ions was found to be higher in Li_2_S_6_ than in Li_2_S_8_. This observation indicates that Li_2_S_6_ exhibits a higher degree of ion mobility and faster transport of Li^+^ ions (refer to Figure 7c,d). The variation in diffusion behavior can be attributed to differences in sulfur species content and their influence on the solvation properties of Li^+^ ions in the electrolyte solution, as discussed in the previous section. Furthermore, we observed that the diffusion coefficient of Li^+^ ions increases considerably with the amount of solvent for Li_2_S_6_, whereas it remains nearly constant for Li_2_S_8_. This observation suggests that the presence of DME or DOL solvents significantly enhances the mobility of Li^+^ ions in the Li_2_S_6_ electrolyte. However, this effect is not as significant in the case of Li_2_S_8_. This finding suggests that a higher concentration of solvent molecules facilitates the movement of Li^+^ ions within the electrolyte solution near the CP_3_ monolayer. The increased diffusion coefficient indicates improved mobility and faster transport of Li^+^ ions within the LiPSs electrolyte.

In the context of Li–S batteries, the presence of Li_2_S_6_ and Li_2_S_8_ species plays a significant role in the electrochemical reactions that take place during the battery’s cycling process.^63^ Thus, it is crucial to maintain a high ionic conductivity in the electrolyte to enable the reversible conversion between sulfur and lithium polysulfides. From Figure 7b, we observed that the ionic conductivity of Li_2_S_6_ electrolyte is higher than that of Li_2_S_8_. The difference in conductivity can be attributed to the structural and chemical dissimilarities between Li_2_S_6_ and Li_2_S_8_, which impact the mobility of Li^+^ ions in its electrolyte. Furthermore, we found that the ionic conductivity of Li_2_S_6_ increased with an increase in the amount of solvent (decrease of S content), whereas for Li_2_S_8_, it remained almost constant for the considered solvent/salt molar fractions, such a trend is experimentally observed.^64,65^ This observation suggests that the presence of DOL or DME solvents has a more significant impact on enhancing Li^+^ ion mobility in Li_2_S_6_ compared to Li_2_S_8_. The higher solvating ability of the solvent molecules in the Li_2_S_6_ electrolyte promotes salt dissociation and facilitates ions transport, resulting in enhanced conductivity.

Furthermore, we calculated the Nernst–Einstein conductivity (σ_ne_) and compared it with the ionic conductivity (σ) as illustrated in Figure 7b. The Nernst–Einstein conductivity is a theoretical estimate based on the Einstein relation. This relation connects the ionic conductivity to the self-diffusion coefficients of ions and temperature. The discrepancy between σ_ne_ and experimental data on ionic conductivity has been extensively discussed in the literature.^54,66^ This discrepancy arises from the assumption made in the Nernst–Einstein (NE) equation that the motion of cations and anions is independent without considering the possibility of ion association in ionic liquids. Thus, σ_ne_ only provides an estimate of conductivity. When ions form pairs or aggregates, they impede ion conductivity instead of actively contributing to it. Although the diffusion coefficient is influenced by the movement of counterions, it does not affect charge transfer or ion conductivity, which can result in an overestimation when using the NE equation. On the other hand, the ionic conductivity obtained through the collective mean square displacement method (Einstein law) is reported to show better agreement with experimental data than the NE approximation. This agreement emphasizes the significance of considering ion motion correlations, which are accounted for in the Einstein law but neglected in the Nernst–Einstein approximation. The collective transport property observed in ionic conductivity provides a better understanding of the influence of ion motion correlations and their impact on conductivity. For Li_2_S_6_, we observed that the ratio of σ_ne_ to σ was approximately 2. This indicates a deviation from ideal behavior. This deviation suggests the presence of additional mechanisms for ionic transport, such as hopping or association/dissociation processes, that contribute to the overall conductivity. The enhanced conductivity of Li_2_S_6_ compared to Li_2_S_8_ can be attributed to additional transport mechanisms that are more prevalent in Li_2_S_6_ due to its higher structural dynamics. In the case of Li_2_S_8_, the ratio of σ_ne_ to σ was approximately 1.63, indicating a relatively smaller deviation from ideal behavior compared to that of Li_2_S_6_. This suggests that ion transport in Li_2_S_8_ is mainly controlled by diffusion, with a minor contribution from the other mechanisms. The observed differences in the ratios of σ_ne_/σ between Li_2_S_6_ and Li_2_S_8_ provide additional evidence of the distinct ionic transport properties of these electrolytes. The higher value for Li_2_S_6_ suggests a more complex mechanism for ion transport, whereas the lower value for Li_2_S_8_indicates a relatively simpler diffusion process. Overall, the calculated ionic conductivity obtained from collective diffusion, denoted as σ, is found to be in agreement with the experimental data.^64,65^ However, the ionic conductivity estimated by the Nernst–Einstein approach, denoted as σ_ne_, overestimates the experimental values.

The uniformly distributed and moderate interaction between CP_3_ and electrolyte components, as illustrated in Figure S4, does not lead to agglomeration or aggregation of electrolyte components near the CP_3_. Therefore, from a macroscopic perspective, CP_3_ does not significantly impact the dynamic properties of the electrolyte. The ionic conductivity ranges from 0.3 to 1.36 mS/cm for Li_2_S_6_ and 0.16 to 0.23 mS/cm for Li_2_S_8_, which are comparable to their values in the bulk electrolyte.^64−66^ These high ionic conductivity values indicate that intermediate compounds can participate effectively in electrochemical processes, facilitating efficient ion transport within the electrolyte. Consequently, this enables faster charging and discharging rates, which are critical for achieving high power densities and enhancing the overall performance of Li–S batteries.

## 4 Conclusion

In summary, the adsorption of S_8_/LiPSs (Li_2_S_*n*_, *n* = 1, 2, 4, 6, and 8) on a CP_3_ monolayer has been systematically investigated from first-principles calculations. Our investigation shows that the CP_3_ monolayer possesses good electrical conductivity, which is crucial to ensure high sulfur utilization. Because of a synergistic dual interaction based on the Li–P and S–P bonds, the CP_3_ monolayer can moderately interact with LiPSs and guide the deposition of Li_2_S with uniform propagation. All of these indicate the key roles of the CP_3_ monolayer in restraining the shuttling of the soluble LiPSs, improving both the rate and cycling performance. In view of the abundance of C and P atoms, this work is expected to open an avenue in searching for the optimal sulfur host materials that have both good electronic conductivity and improved LiPS affinity.

## Supporting Information Available

Lithium-ion diffusion barrier and Li_2_S decomposition on 2D CP_3_ catalyst surface curve; statistical distribution of the coordination number curves; radial distribution function and coordination number average profiles of P atoms around a single Li^+^ ion curve (PDF)

## References

[ref1] Chen X.; Hou T.; Persson K. A.; Zhang Q. Combining Theory and Experiment in Lithium–Sulfur Batteries: Current Progress and Future Perspectives. Mater. Today 2019, 22, 142–158. 10.1016/j.mattod.2018.04.007.

[ref2] Xu Z.-L.; Lin S.; Onofrio N.; Zhou L.; Shi F.; Lu W.; Kang K.; Zhang Q.; Lau S. P. Exceptional Catalytic Effects of Black Phosphorus Quantum Dots in Shuttling-free Lithium Sulfur Batteries. Nat. Commun. 2018, 9, 4164 10.1038/s41467-018-06629-9. 30301957 PMC6177446

[ref3] Lemaalem M.; Khossossi N.; Bouder G.; Dey P.; Carbonnière P. Graphyne-based Membrane As a Promising Candidate for Li-battery Electrodes Protection: Insight from Atomistic Simulations. J. Power Sources 2023, 581, 233482 10.1016/j.jpowsour.2023.233482.

[ref4] Singh D.; Shukla V.; Khossossi N.; Ainane A.; Ahuja R. Harnessing the Unique Properties of Mxenes for Advanced Rechargeable Batteries. J. Phys.: Energy 2021, 3, 012005 10.1088/2515-7655/abceac.

[ref5] Cheng X.-B.; Zhang R.; Zhao C.-Z.; Zhang Q. Toward Safe Lithium Metal Anode in Rechargeable Batteries: a Review. Chem. Rev. 2017, 117, 10403–10473. 10.1021/acs.chemrev.7b00115. 28753298

[ref6] Wang S.-H.; Yin Y.-X.; Zuo T.-T.; Dong W.; Li J.-Y.; Shi J.-L.; Zhang C.-H.; Li N.-W.; Li C.-J.; Guo Y.-G. Stable Li Metal Anodes via Regulating Lithium Plating/stripping in Vertically Aligned Microchannels. Adv. Mater. 2017, 29, 1703729 10.1002/adma.201703729. 28891207

[ref7] Bruce P. G.; Freunberger S. A.; Hardwick L. J.; Tarascon J.-M. Li–O_2_ and Li–S Batteries with High Energy Storage. Nature Mater. 2012, 11, 19–29. 10.1038/nmat3191. 22169914

[ref8] Zhou S.; Shi J.; Liu S.; Li G.; Pei F.; Chen Y.; Deng J.; Zheng Q.; Li J.; Zhao C.; et al. Visualizing Interfacial Collective Reaction Behaviour of Li–S Batteries. Nature 2023, 621, 75–81. 10.1038/s41586-023-06326-8. 37673990

[ref9] Evers S.; Yim T.; Nazar L. F. Understanding the Nature of Absorption/adsorption in Nanoporous Polysulfide Sorbents for the Li–S Battery. J. Phys. Chem. C 2012, 116, 19653–19658. 10.1021/jp304380j.

[ref10] Khossossi N.; Singh D.; Essaoudi I.; Ahuja R.; Ainane A. Unveiling the Catalytic Potential of Two-dimensional Boron Nitride in Lithium-Sulfur Batteries. Chem. Eng. J. 2024, 479, 147518 10.1016/j.cej.2023.147518.

[ref11] Chu H.; Noh H.; Kim Y.-J.; Yuk S.; Lee J.-H.; Lee J.; Kwack H.; Kim Y.; Yang D.-K.; Kim H.-T. Achieving Three-dimensional Lithium Sulfide Growth in Lithium-Sulfur Batteries Using High-donor-number Anions. Nat. Commun. 2019, 10, 188 10.1038/s41467-018-07975-4. 30643115 PMC6331553

[ref12] Deng C.; Wang Z.; Wang S.; Yu J. Inhibition of Polysulfide Diffusion in Lithium–Sulfur Batteries: Mechanism and Improvement Strategies. J. Mater. Chem. A 2019, 7, 12381–12413. 10.1039/C9TA00535H.

[ref13] Li J.; Qu Y.; Chen C.; Zhang X.; Shao M. Theoretical Investigation on Lithium Polysulfide Adsorption and Conversion for High-performance Li–S Batteries. Nanoscale 2021, 13, 15–35. 10.1039/D0NR06732F. 33325951

[ref14] Khossossi N.; Panda P. K.; Singh D.; Shukla V.; Mishra Y. K.; Essaoudi I.; Ainane A.; Ahuja R. Rational Design of 2D h-BAs Monolayer as Advanced Sulfur Host for High Energy Density Li–S Batteries. ACS Appl. Energy Mater. 2020, 3, 7306–7317. 10.1021/acsaem.0c00492.

[ref15] Liu D.; Zhang C.; Zhou G.; Lv W.; Ling G.; Zhi L.; Yang Q.-H. Catalytic Effects in Lithium–sulfur Batteries: Promoted Sulfur Transformation and Reduced Shuttle Effect. Adv. Sci. 2018, 5, 1700270 10.1002/advs.201700270. PMC5770674 29375960

[ref16] Wang R.; Luo C.; Wang T.; Zhou G.; Deng Y.; He Y.; Zhang Q.; Kang F.; Lv W.; Yang Q.-H. Bidirectional Catalysts for Liquid–solid Redox Conversion in Lithium–Sulfur Batteries. Adv. Mater. 2020, 32, 2000315 10.1002/adma.202000315. 32627911

[ref17] Manthiram A.; Chung S.-H.; Zu C. Lithium–sulfur Batteries: Progress and Prospects. Adv. Mater. 2015, 27, 1980–2006. 10.1002/adma.201405115. 25688969

[ref18] Zhang L.; Wang Y.; Niu Z.; Chen J. Advanced Nanostructured Carbon-based Materials for Rechargeable Lithium-sulfur Batteries. Carbon 2019, 141, 400–416. 10.1016/j.carbon.2018.09.067.

[ref19] Wu Q.; Zhou X.; Xu J.; Cao F.; Li C. Carbon-based Derivatives from Metal-organic Frameworks As Cathode Hosts for Li–S Batteries. J. Energy Chem. 2019, 38, 94–113. 10.1016/j.jechem.2019.01.005.

[ref20] Pang Q.; Liang X.; Kwok C. Y.; Nazar L. F. Advances in Lithium–sulfur Batteries Based on Multifunctional Cathodes and Electrolytes. Nat. Energy 2016, 1, 16132 10.1038/nenergy.2016.132.

[ref21] Liang X.; Hart C.; Pang Q.; Garsuch A.; Weiss T.; Nazar L. F. A Highly Efficient Polysulfide Mediator for Lithium–Sulfur Batteries. Nat. Commun. 2015, 6, 5682 10.1038/ncomms6682. 25562485

[ref22] Seh Z. W.; Li W.; Cha J. J.; Zheng G.; Yang Y.; McDowell M. T.; Hsu P.-C.; Cui Y. Sulphur–TiO_2_ Yolk–shell Nanoarchitecture with Internal Void Space for Long-cycle Lithium–Sulphur Batteries. Nat. Commun. 2013, 4, 1331 10.1038/ncomms2327. 23299881

[ref23] Zhou L.; Danilov D. L.; Eichel R.-A.; Notten P. H. Host Materials Anchoring Polysulfides in Li–S Batteries Reviewed. Adv. Energy Mater. 2021, 11, 2001304 10.1002/aenm.202001304.

[ref24] Abbasi N. M.; Xiao Y.; Zhang L.; Peng L.; Duo Y.; Wang L.; Yin P.; Ge Y.; Zhu H.; Zhang B.; et al. Heterostructures of Titanium-based MXenes in Energy Conversion and Storage Devices. J. Mater. Chem. C 2021, 9, 8395–8465. 10.1039/D1TC00327E.

[ref25] Li Y.; Ye D.; Liu W.; Shi B.; Guo R.; Zhao H.; Pei H.; Xu J.; Xie J. A MnO_2_/graphene Oxide/multi-walled Carbon Nanotubes-sulfur Composite with Dual-efficient Polysulfide Adsorption for Improving Lithium-Sulfur Batteries. ACS Appl. Mater. Interfaces 2016, 8, 28566–28573. 10.1021/acsami.6b04270. 27472481

[ref26] Yu X.; Zhu X.; Pei Z.; Li Y.; Li C.; Sui Z. Nitrogen-doped Porous Graphene/MnO_2_ Composite As Sulfur Hosts for Lithium-Sulfur Batteries. Diamond Relat. Mater. 2021, 118, 108497 10.1016/j.diamond.2021.108497.

[ref27] Liu Y.; Zhang Y.; Liu Y.; Zhu J.; Ge Z.; Li Z.; Chen Y. Super Heating/cooling Rate Enabled by Microwave Shock on Polymeric Graphene Foam for High Performance Lithium–Sulfur Batteries. Carbon 2021, 173, 809–816. 10.1016/j.carbon.2020.11.061.

[ref28] Sun K.; Fu M.; Xie Z.; Su D.; Zhong H.; Bai J.; Dooryhee E.; Gan H. Improvement of Li-S Battery Electrochemical Performance with 2d TiS_2_ Additive. Electrochim. Acta 2018, 292, 779–788. 10.1016/j.electacta.2018.09.191.

[ref29] Al Salem H.; Chitturi V. R.; Babu G.; Santana J. A.; Gopalakrishnan D.; Reddy Arava L. M. Stabilizing Polysulfide-shuttle in a Li–S Battery Using Transition Metal Carbide Nanostructures. RSC Adv. 2016, 6, 110301–110306. 10.1039/C6RA22434B.

[ref30] Mahankali K.; Thangavel N. K.; Gopchenko D.; Arava L. M. R. Atomically Engineered Transition Metal Dichalcogenides for Liquid Polysulfide Adsorption and Their Effective Conversion in Li-S Batteries. ACS Appl. Mater. Interfaces 2020, 12, 27112–27121. 10.1021/acsami.0c04281. 32432451

[ref31] Li H.; Sun L.; Zhang Y.; Tan T.; Wang G.; Bakenov Z. Enhanced Cycle Performance of Li/S Battery with the Reduced Graphene Oxide/activated Carbon Functional Interlayer. J. Energy Chem. 2017, 26, 1276–1281. 10.1016/j.jechem.2017.09.009.

[ref32] Park G. D.; Lee J.; Piao Y.; Kang Y. C. Mesoporous Graphitic Carbon-TiO_2_ Composite Microspheres Produced by a Pilot-scale Spray-drying Process As an Efficient Sulfur Host Material for Li-S Batteries. Chem. Eng. J. 2018, 335, 600–611. 10.1016/j.cej.2017.11.021.

[ref33] Gullman J.; Olofsson O. The Crystal Structure of SnP_3_ and a Note on the Crystal Structure of GeP_3_. J. Solid State Chem. 1972, 5, 441–445. 10.1016/0022-4596(72)90091-6.

[ref34] Häggström L.; Gullman J.; Ericsson T.; Wäppling R. Mössbauer Study of Tin Phosphides. J. Solid State Chem. 1975, 13, 204–207. 10.1016/0022-4596(75)90120-6.

[ref35] Donohue P.; Young H. Synthesis, Structure, and Superconductivity of New High Pressure Phases in the Systems GeP and GeAs. J. Solid State Chem. 1970, 1, 143–149. 10.1016/0022-4596(70)90005-8.

[ref36] Sun S.; Meng F.; Wang H.; Wang H.; Ni Y. Novel Two-dimensional Semiconductor SnP_3_: High Stability, Tunable Bandgaps and High Carrier Mobility Explored Using First-principles Calculations. J. Mater. Chem. A 2018, 6, 11890–11897. 10.1039/C8TA02494D.

[ref37] Jing Y.; Ma Y.; Li Y.; Heine T. GeP_3_: a Small Indirect Band Gap 2d Crystal with High Carrier Mobility and Strong Interlayer Quantum Confinement. Nano Lett. 2017, 17, 1833–1838. 10.1021/acs.nanolett.6b05143. 28125237

[ref38] Feng L.-P.; Li A.; Wang P.-C.; Liu Z.-T. Novel Two-dimensional Semiconductor SnP_3_ with High Carrier Mobility, Good Light Absorption, and Strong Interlayer Quantum Confinement. J. Phys. Chem. C 2018, 122, 24359–24367. 10.1021/acs.jpcc.8b06211.

[ref39] Ramzan M. S.; Bacic V.; Jing Y.; Kuc A. Electronic Properties of a New Family of Layered Materials from Groups 14 and 15: First-principles Simulations. J. Phys. Chem. C 2019, 123, 25470–25476. 10.1021/acs.jpcc.9b07068.

[ref40] Kar M.; Sarkar R.; Pal S.; Sarkar P. Two-dimensional CP_3_ Monolayer and Its Fluorinated Derivative with Promising Electronic and Optical Properties: a Theoretical Study. Phys. Rev. B 2020, 101, 195305 10.1103/PhysRevB.101.195305.

[ref41] Kresse G.; Furthmüller J. Efficient Iterative Schemes for Ab Initio Total-energy Calculations Using a Plane-wave Basis Set. Phys. Rev. B 1996, 54, 11169 10.1103/PhysRevB.54.11169. 9984901

[ref42] Perdew J. P.; Burke K.; Ernzerhof M. Generalized Gradient Approximation Made Simple. Phys. Rev. Lett. 1996, 77, 3865 10.1103/PhysRevLett.77.3865. 10062328

[ref43] Monkhorst H. J.; Pack J. D. Special Points for Brillouin-zone Integrations. Phys. Rev. B 1976, 13, 5188 10.1103/PhysRevB.13.5188.

[ref44] Henkelman G.; Arnaldsson A.; Jónsson H. A Fast and Robust Algorithm for Bader Decomposition of Charge Density. Comput. Mater. Sci. 2006, 36, 354–360. 10.1016/j.commatsci.2005.04.010.

[ref45] Dodda L. S.; Cabeza de Vaca I.; Tirado-Rives J.; Jorgensen W. L. Ligpargen Web Server: an Automatic OPLS-AA Parameter Generator for Organic Ligands. Nucleic Acids Res. 2017, 45, W331–W336. 10.1093/nar/gkx312. 28444340 PMC5793816

[ref46] Siu S. W. I.; Pluhackova K.; Böckmann R. A. Optimization of the OPLS-AA Force Field for Long Hydrocarbons. J. Chem. Theory Comput. 2012, 8, 1459–1470. 10.1021/ct200908r. 26596756

[ref47] Canongia Lopes J. N.; Padua J. N. CL&P: A Generic and Systematic Force Field for Ionic Liquids Modeling. Theor. Chem. Acc. 2012, 131, 1129 10.1007/s00214-012-1129-7.

[ref48] Rajput N. N.; Murugesan V.; Shin Y.; Han K. S.; Lau K. C.; Chen J.; Liu J.; Curtiss L. A.; Mueller K. T.; Persson K. A. Elucidating the Solvation Structure and Dynamics of Lithium Polysulfides Resulting from Competitive Salt and Solvent Interactions. Chem. Mater. 2017, 29, 3375–3379. 10.1021/acs.chemmater.7b00068.

[ref49] Thompson A. P.; Aktulga H. M.; Berger R.; Bolintineanu D. S.; Brown W. M.; Crozier P. S.; in ’t Veld P. J.; Kohlmeyer A.; Moore S. G.; Nguyen T. D.; Shan R.; Stevens M. J.; Tranchida J.; Trott C.; Plimpton S. J. Lammps - a Flexible Simulation Tool for Particle-based Materials Modeling at the Atomic, Meso, and Continuum Scales. Comput. Phys. Commun. 2022, 271, 108171 10.1016/j.cpc.2021.108171.

[ref50] Jewett A. I.; Stelter D.; Lambert J.; Saladi S. M.; Roscioni O. M.; Ricci M.; Autin L.; Maritan M.; Bashusqeh S. M.; Keyes T.; Dame R. T.; Shea J.-E.; Jensen G. J.; Goodsell D. S. Moltemplate: a Tool for Coarse-grained Modeling of Complex Biological Matter and Soft Condensed Matter Physics. J. Mol. Biol. 2021, 433, 166841 10.1016/j.jmb.2021.166841. 33539886 PMC8119336

[ref51] Lemaalem M.; Carbonniere P. Effects of Solvents on Li+ Distribution and Dynamics in PVDF/LiFSI Solid Polymer Electrolytes: an All-atom Molecular Dynamics Simulation Study. Solid State Ionics 2023, 399, 116304 10.1016/j.ssi.2023.116304.

[ref52] Lemaalem M.; Carbonnière P. Tunable Properties of Poly(vinylidene fluoride)-derived Polymers for Advancing Battery Performance and Enabling Diverse Applications. Polymer 2023, 283, 126218 10.1016/j.polymer.2023.126218.

[ref53] Lemaalem M.; Hadrioui N.; Derouiche A.; Ridouane H. Structure and Dynamics of Liposomes Designed for Drug Delivery: Coarse-grained Molecular Dynamics Simulations to Reveal the Role of Lipopolymer Incorporation. RSC Adv. 2020, 10, 3745–3755. 10.1039/C9RA08632C. 35492626 PMC9048902

[ref54] Basouli H.; Mozaffari F.; Eslami H. Atomistic Insights into Structure, Ion-pairing and Ionic Conductivity of 1-ethyl-3-methylimidazolium Methylsulfate [Emim][MeSO_4_] Ionic Liquid from Molecular Dynamics Simulation. J. Mol. Liq. 2021, 331, 115803 10.1016/j.molliq.2021.115803.

[ref55] Cheng Z.; Zhang X.; Zhang H.; Gao J.; Liu H.; Yu X.; Dai X.; Liu G.; Chen G. Prediction of Two-dimensional CP_3_ as a Promising Electrode Material with a Record-high Capacity for Na Ions. Nanoscale Adv. 2020, 2, 5271–5279. 10.1039/D0NA00746C. 36132047 PMC9418581

[ref56] Zhang D.; Duan T.; Luo Y.; Liu S.; Zhang W.; He Y.; Zhu K.; Huang L.; Yang Y.; Yu R.; et al. Oxygen Defect-rich WO_3-x_ –W_3_N_4_ Mott–schottky Heterojunctions Enabling Bidirectional Catalysis for Sulfur Cathode. Adv. Funct. Mater. 2023, 33, 2306578 10.1002/adfm.202306578.

[ref57] Li C.; Ge W.; Qi S.; Zhu L.; Huang R.; Zhao M.; Qian Y.; Xu L. Manipulating Electrocatalytic Polysulfide Redox Kinetics by 1d Core–shell Like Composite for Lithium–sulfur Batteries. Adv. Energy Mater. 2022, 12, 2103915 10.1002/aenm.202103915.

[ref58] Ma C.; Zhang Y.; Feng Y.; Wang N.; Zhou L.; Liang C.; Chen L.; Lai Y.; Ji X.; Yan C.; et al. Engineering Fe–N Coordination Structures for Fast Redox Conversion in Lithium–sulfur Batteries. Adv. Mater. 2021, 33, 2100171 10.1002/adma.202100171. 34145629

[ref59] Khossossi N.; Singh D.; Essaoudi I.; Ahuja R.; Ainane A. Unveiling the Catalytic Potential of Two-dimensional Boron Nitride in Lithium–sulfur Batteries. Chem. Eng. J. 2024, 479, 147518 10.1016/j.cej.2023.147518.

[ref60] Korff D.; Colclasure A. M.; Ha Y.; Smith K. A.; DeCaluwe S. C. Pathways Toward High-energy Li-sulfur Batteries, Identified via Multi-reaction Chemical Modeling. J. Electrochem. Soc. 2022, 169, 010520 10.1149/1945-7111/ac4541.

[ref61] Liu Y.; Elias Y.; Meng J.; Aurbach D.; Zou R.; Xia D.; Pang Q. Electrolyte Solutions Design for Lithium-sulfur Batteries. Joule 2021, 5, 2323–2364. 10.1016/j.joule.2021.06.009.

[ref62] Lang S.; Feng X.; Seok J.; Yang Y.; Krumov M. R.; Molina Villarino A.; Lowe M. A.; Yu S.-H.; Abruña H. D. Lithium–sulfur Redox: Challenges and Opportunities. Curr. Opin. Electrochem. 2021, 25, 100652 10.1016/j.coelec.2020.100652.

[ref63] Lei J.; Liu T.; Chen J.; Zheng M.; Zhang Q.; Mao B.; Dong Q. Exploring and Understanding the Roles of Li_2_S_n_ and the Strategies to Beyond Present Li-S Batteries. Chem 2020, 6, 2533–2557. 10.1016/j.chempr.2020.06.032.

[ref64] Fan F. Y.; Pan M. S.; Lau K. C.; Assary R. S.; Woodford W. H.; Curtiss L. A.; Carter W. C.; Chiang Y.-M. Solvent Effects on Polysulfide Redox Kinetics and Ionic Conductivity in Lithium-Sulfur Batteries. J. Electrochem. Soc. 2016, 163, A3111 10.1149/2.1181614jes.

[ref65] Park C.; Ronneburg A.; Risse S.; Ballauff M.; Kanduc M.; Dzubiella J. Structural and Transport Properties of Li/s Battery Electrolytes: Role of the Polysulfide Species. J. Phys. Chem. C 2019, 123, 10167–10177. 10.1021/acs.jpcc.8b10175.

[ref66] Park C.; Kanduč M.; Chudoba R.; Ronneburg A.; Risse S.; Ballauff M.; Dzubiella J. Molecular Simulations of Electrolyte Structure and Dynamics in Lithium–sulfur Battery Solvents. J. Power Sources 2018, 373, 70–78. 10.1016/j.jpowsour.2017.10.081.

