# Supplementary material for: 2D Carbon Phosphide for Trapping Sulfur in Rechargeable Li–S Batteries: Structure Design and Interfacial Chemistry
Source: ACS Appl Mater Interfaces. 2024 Dec 16;17(1):930–42. doi: 10.1021/acsami.4c15372 (PMC11783354; doi:10.1021/acsami.4c15372)
Supplement: Supplementary file 1 — am4c15372_si_001.pdf [file am4c15372_si_001.pdf]

Supporting Information

2D Carbon Phosphide for Trapping Sulfur in Rechargeable Li–S Batteries: Structure Design and Interfacial Chemistry

Nabil Khossossi[a,*], *Mohammed Lemaalem*[b,c], *Talha Zafer*[a,d], *Abdelfattah Mahmoud*[e], *and Poulumi Dey*[a]

[a]Department of Materials Science and Engineering, Faculty of Mechanical Engineering, Delft University of Technology, Mekelweg 2, Delft, 2628 CD, The Netherlands

[b]Department of Chemical Engineering, University of Illinois Chicago, Chicago, IL60608, USA

[c]Materials Science Division, Argonne National Laboratory, Lemont, IL 60439, USA

[d]Vocational School of Health Services, Sakarya University, 54050, Sakarya, Turkey

[e]GREENMAT, CESAM, Institute of Chemistry B6, University of Liège, 4000 Liège, Belgium

*Corresponding author: n.khossossi@tudelft.nl

CONTENTS

1. **Figure S1.** Lithium-ion diffusion barrier and Li_2S decomposition on the CP_3 catalyst surface.

2. **Figure S2.** Coordination Number (N(r)) distributions of Li^+ for Li_2S_6/DME and Li_2S_6/DOL electrolytes near CP_3.

3. **Figure S3.** Coordination Number (N(r)) distributions of Li^+ for Li_2S_8/DME and Li_2S_8/DOL electrolytes near CP_3.

4. **Figure S4.** Radial Distribution Function (g(r)) and Coordination Number (N(r)) profiles of Phosphorus atoms and Sulfur anions around CP_3.

Figure S1

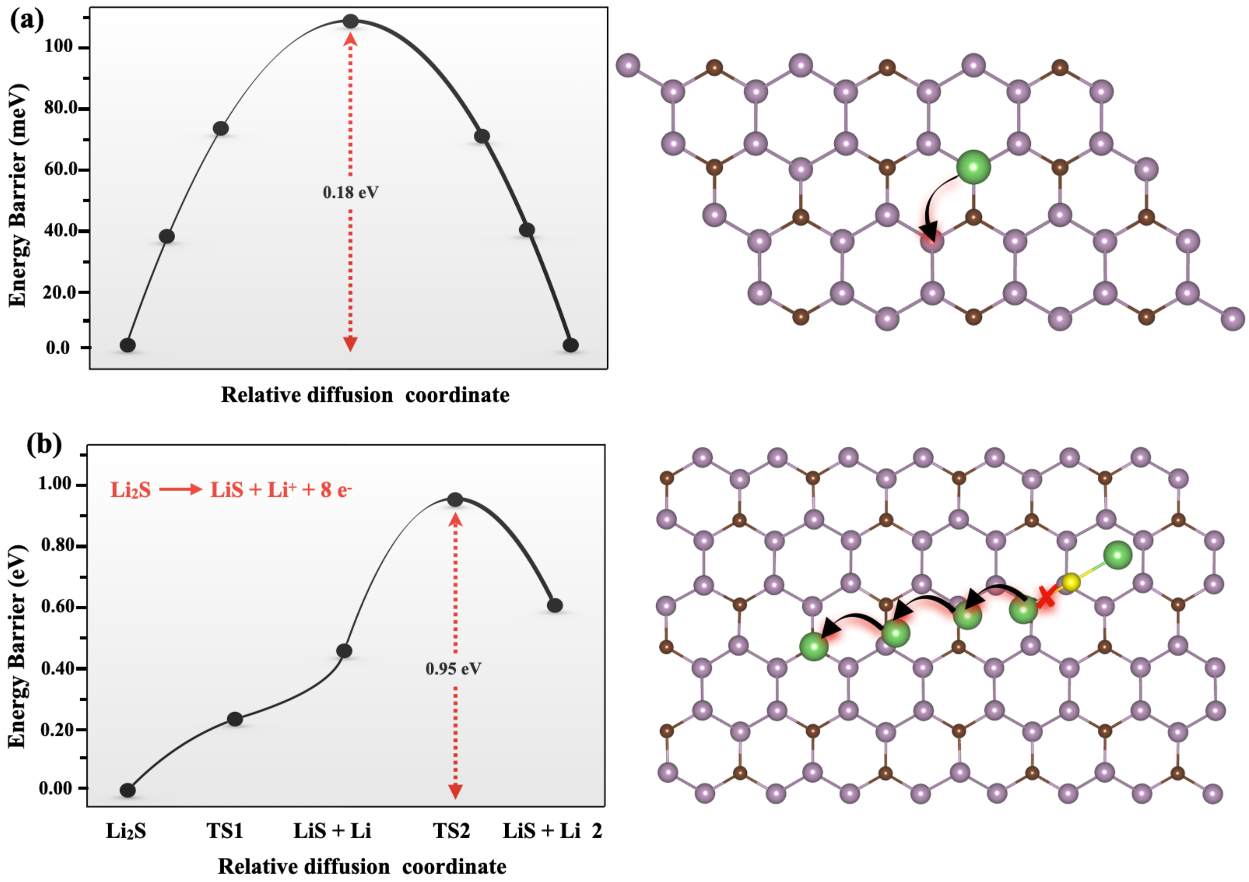

Figure 1: (a) Lithium-ion diffusion barrier and (b) Li_2S decomposition on the 2D CP_3 catalyst surface. The figure demonstrates the catalyst's efficacy in facilitating lithium-ion diffusion and sulfur decomposition.

Figure S2

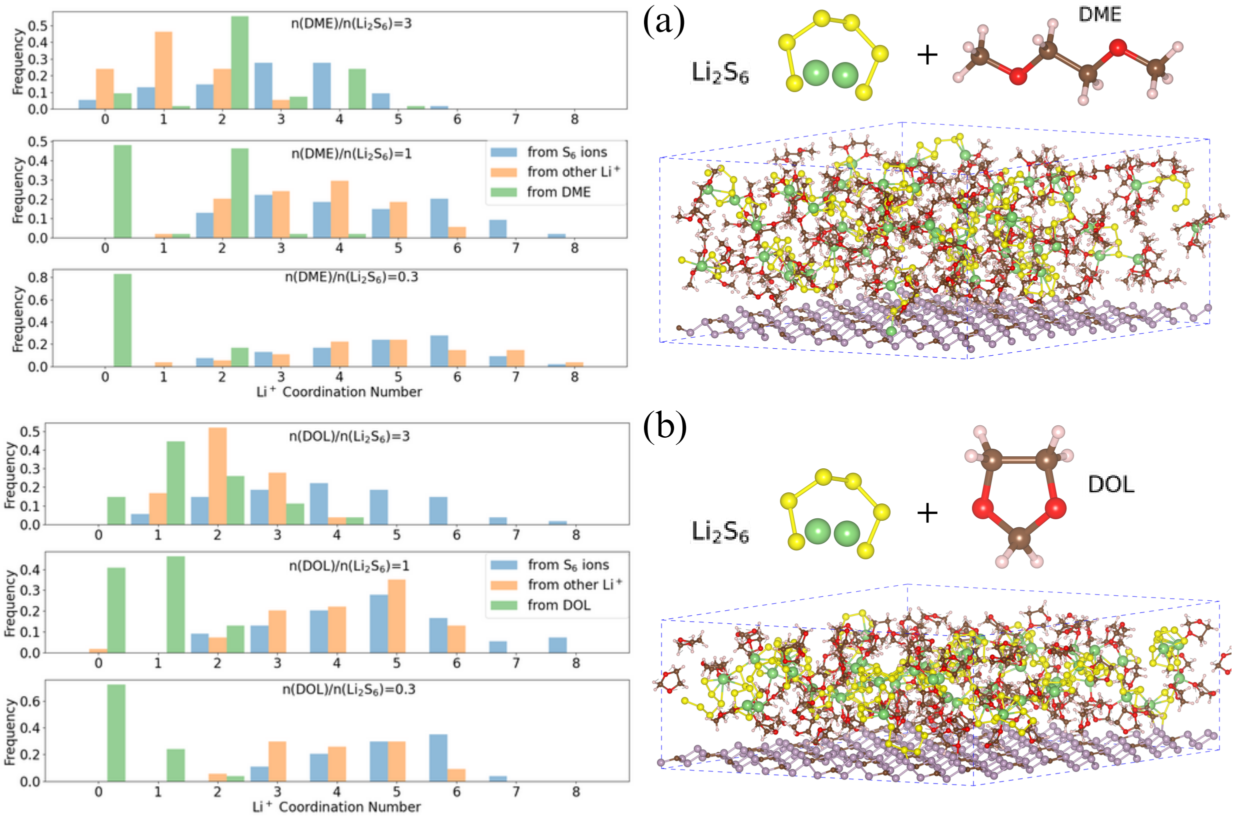

Figure 2: Statistical distribution of the Coordination Number (N(r)) values of a tagged Li^+ from S_6 anions, solvent Oxygen atoms (O), and surrounding Li^+ ions. The results are shown for the simulated electrolyte systems Li_2S_6/DME ((a)) and Li_2S_6/DOL ((b)) near the CP_3 monolayer with varying solvent amounts.

Figure S3

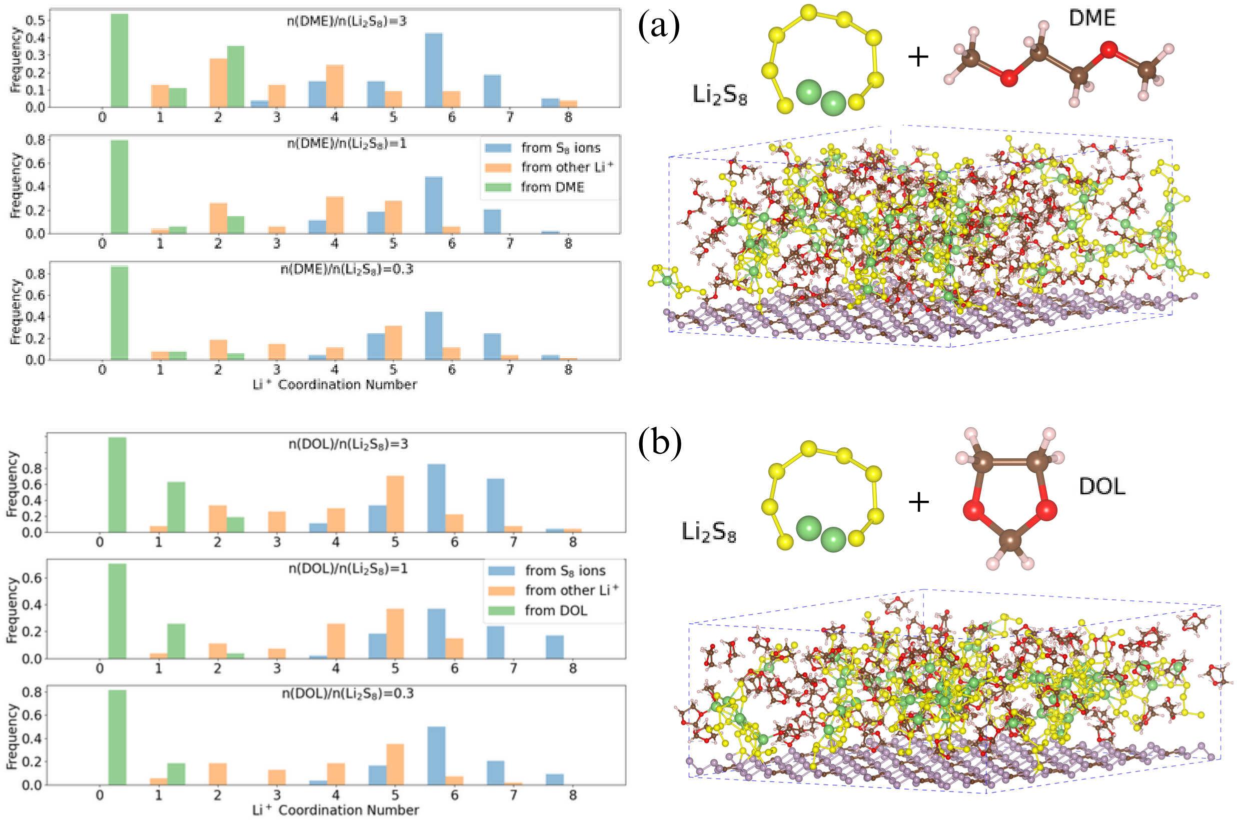

Figure 3: Statistical distribution of the Coordination Number (N(r)) values of a tagged Li^+ from S_8 anions, solvent Oxygen atoms (O), and surrounding Li^+ ions. Results are compared for the simulated electrolyte systems Li_2S_8/DME ((a)) and Li_2S_8/DOL ((b)) near the CP_3 monolayer with varying solvent amounts.

Figure S4

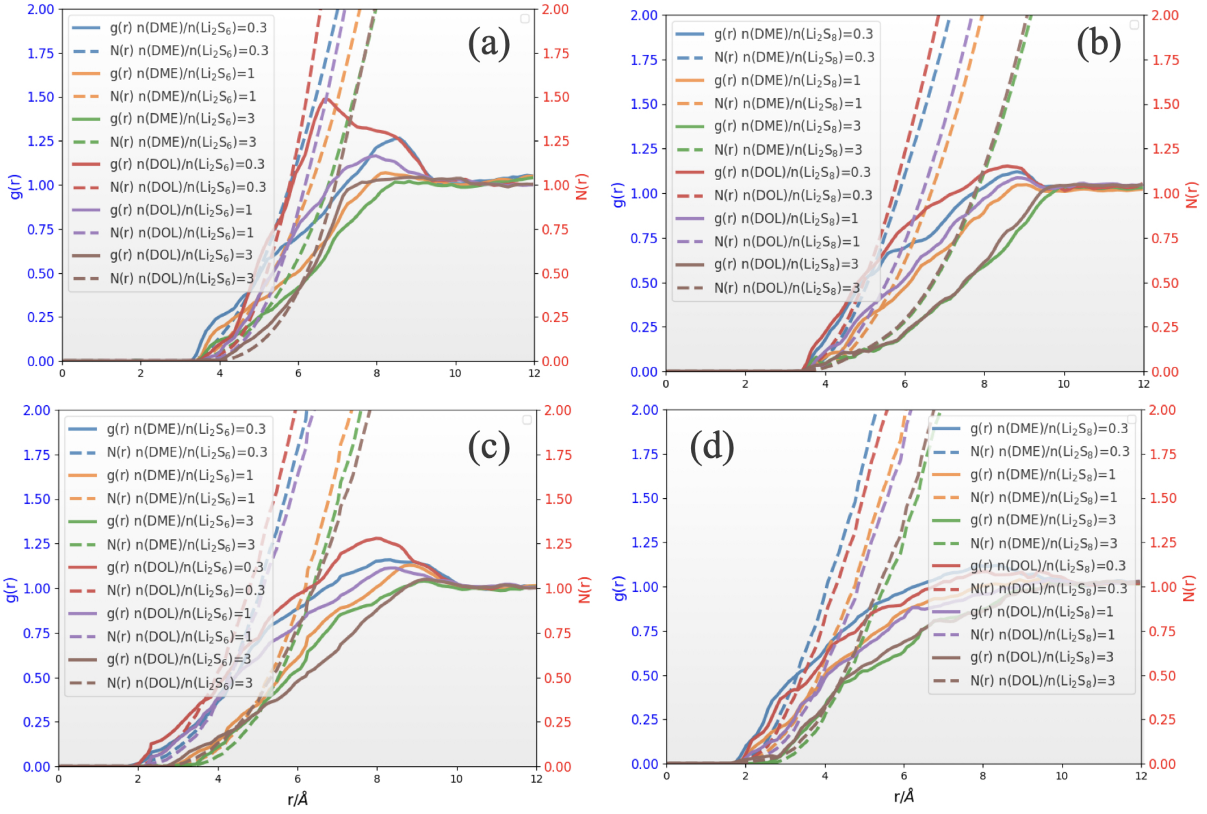

Figure 4: Comparison of Radial Distribution Function (g(r)) and Coordination Number (N(r)) average profiles of Phosphorus atoms (P) around a single Li^+ ion in simulated electrolytes near the CP_3 surface. (a,b) depict Li_2S_6/DME and Li_2S_6/DOL electrolytes, while (c,d) illustrate Li_2S_8/DME and Li_2S_8/DOL electrolytes. The profiles also show the surrounding Sulfur anions $S^{-\delta}$ with varying partial charges.
